# The use of GRADE-CERQual in qualitative evidence synthesis: an evaluation of fidelity and reporting

**DOI:** 10.1186/s12961-023-00999-3

**Published:** 2023-07-25

**Authors:** Megan Wainwright, Rana Islamiah Zahroh, Özge Tunçalp, Andrew Booth, Meghan A. Bohren, Jane Noyes, Weilong Cheng, Heather Munthe-Kaas, Simon Lewin

**Affiliations:** 1 Department of Anthropology, Faculty of Social Sciences and Health, Durham University, South Road, Durham, United Kingdom; 2 Gender and Women’s Health Unit, School of Population and Global Health, Centre for Health Equity, The University of Melbourne, Carlton, VIC Australia; 3 UNDP/UNFPA/UNICEF/WHO/World Bank Special Programme of Research, Development and Research Training in Human Reproduction (HRP), Department of Sexual and Reproductive Health and Research, World Health Organization, Geneva, Switzerland; 4 Faculty of Medicine, Dentistry and Health, School of Health and Related Research (ScHARR), University of Sheffield, Sheffield, United Kingdom; 5 School of Medical and Health Sciences, Bangor University, Bangor, Wales United Kingdom; 6 Melbourne School of Population and Global Health, Centre for Epidemiology and Biostatistics, The University of Melbourne, Carlton, VIC Australia; 7 The Centre for Epidemic Interventions Research, Norwegian Institute of Public Health, Oslo, Norway; 8 Division of Health Services and Centre for Epidemic Interventions Research (CEIR), Norwegian Institute of Public Health, Oslo, Norway; 9 Health Systems Research Unit, South African Medical Research Council, Cape Town, South Africa; 10 Department of Health Sciences Ålesund, Norwegian University of Science and Technology (NTNU), Ålesund, Norway

**Keywords:** Decision-making, Evaluation, Fidelity, GRADE-CERQual, Methodology, Qualitative evidence synthesis, Qualitative research, Reporting, Systematic review

## Abstract

**Background:**

GRADE-CERQual (Confidence in the Evidence from Reviews of Qualitative Research) is a methodological approach to systematically and transparently assess how much confidence decision makers can place in individual review findings from qualitative evidence syntheses. The number of reviews applying GRADE-CERQual is rapidly expanding in guideline and other decision-making contexts. The objectives of this evaluation were, firstly, to describe the uptake of GRADE-CERQual in qualitative evidence synthesis by review authors and, secondly, to assess both reporting of and fidelity to the approach.

**Methods:**

The evaluation had two parts. Part 1 was a citation analysis and descriptive overview of the literature citing GRADE-CERQual. Authors worked together to code and chart the citations, first by title and abstract and second by full text. Part 2 was an assessment and analysis of fidelity to, and reporting of, the GRADE-CERQual approach in included reviews. We developed fidelity and reporting questions and answers based on the most recent guidance for GRADE-CERQual and then used NVivo12 to document assessments in a spreadsheet and code full-text PDF articles for any concerns that had been identified. Our assessments were exported to Excel and we applied count formulae to explore patterns in the data. We employed a qualitative content analysis approach in NVivo12 to sub-coding all the data illustrating concerns for each reporting and fidelity criteria.

**Results:**

233 studies have applied the GRADE-CERQual approach, with most (*n* = 225, 96.5%) in the field of health research. Many studies (*n* = 97/233, 41.6%) were excluded from full fidelity and reporting assessment because they demonstrated a serious misapplication of GRADE-CERQual, for example interpreting it as a quality appraisal tool for primary studies or reviews. For the remaining studies that applied GRADE-CERQual to assess confidence in review findings, the main areas of reporting concern involved terminology, labelling and completeness. Fidelity concerns were identified in more than half of all studies assessed.

**Conclusions:**

GRADE-CERQual is being used widely within qualitative evidence syntheses and there are common reporting and fidelity issues. Most of these are avoidable and we highlight these as gaps in knowledge and guidance for applying the GRADE-CERQual approach.

**Supplementary Information:**

The online version contains supplementary material available at 10.1186/s12961-023-00999-3.

## Background

GRADE-CERQual (Confidence in the Evidence from Reviews of Qualitative Research) is a methodological approach for systematically and transparently assessing how much confidence decision makers can place in individual review findings from qualitative evidence syntheses (QES) (also called systematic reviews of qualitative research). The GRADE-CERQual approach first emerged during the development of the OptimizeMNH guidelines (2012) [1], the first World Health Organization (WHO) guideline to formally incorporate QES in its evidence base, and from which the first QES was published in the Cochrane Library [2]. The authors of the first QES, together with the GRADE Working Group (https://www.gradeworkinggroup.org), the Cochrane Qualitative and Implementation Methods Group (https://methods.cochrane.org/qi/), and the WHO Department of Reproductive Health and Research, developed the first version of GRADE-CERQual that included only two components – Methodological Limitations and Coherence. The two component GRADE-CERQual was disseminated via a conference presentation [3] and published systematic reviews [4–7]. The two component version was very soon replaced by the four component version of GRADE-CERQual which added the relevance and adequacy components to the two component version. It is described in Chapter 21 in the Cochrane handbook [8, 9]. A fifth potential component (dissemination bias) is currently under development [10–12] but not yet included in the GRADE-CERQual approach.

Over the course of its development, the originators of GRADE-CERQual used a human-centred design approach to further develop the current version, including stakeholder engagement and consensus building sessions in 2014 and 2015, piloting the approach in a number of reviews, gathering feedback via online surveys sent to review teams who had applied the approach, and developing a global network of collaborators across diverse institutions to support further consensus development. A GRADE-CERQual coordinating team (12 members) and project group (more than 200 members) were established within the wider GRADE Project Group to continue developing and testing the methods for assessing the confidence in qualitative evidence [13].

The first official guidance on the four component GRADE-CERQual approach was published in 2015 in *PLOS Medicine* [9]. In 2018, the project group published more detailed guidance to help review authors operationalise GRADE-CERQual in their reviews. The guidance is published as a seven paper supplement in *Implementation Science* that includes a paper for each component and the overall assessment [10, 14–19]. Beyond the formally published guidance, and adoption by leading global systematic review organisations and guideline producers, dissemination of the GRADE-CERQual approach has included monthly introductory and question and answer webinars, a newsletter, mentorship of review teams, a shared drive of templates to assist users in applying the approach, regular in-person and virtual training workshops, a train the trainer workshop, and translation of the approach into other languages [20–23]. Recent years have seen development of a free online platform to assist review authors with applying the approach—the GRADE-CERQual interactive Summary of Qualitative Findings (iSoQ) tool [24].

In summary, GRADE-CERQual is a method applied in the late stages of a qualitative evidence synthesis, following completion of analysis and synthesis and after review findings have been drafted. Findings are written-up in detail in the body of the manuscript, and shorter summaries of review findings are drafted for inclusion in the GRADE-CERQual Evidence Profile and Summary of Qualitative Findings (SoQF) tables [15], that form two key outputs from the approach. These two tables incorporate synthesis findings, confidence assessments, explanations and contributing studies in a user-friendly format for decision makers. First, review authors assess each of the four GRADE-CERQual components by identifying any concerns that could reduce their confidence in the review finding. They make a judgement as to whether there are no or very minor, minor, moderate or serious concerns for each component and record each judgement in the Evidence Profile table, accompanied by an explanation. Once each individual component is assessed, the review authors proceed to an overall assessment of confidence, considering assessments made for all four components, and decide whether any identified concerns lower their confidence in the review finding. The review authors start from the assumption that confidence in the finding is high, and then, based on identified concerns for each component, justify downgrading the level of confidence to moderate, low or very low. The guidance papers provide step-by-step instructions for assessing each component and for making the overall assessment [14–19].

The number of reviews applying GRADE-CERQual is expanding rapidly, especially as the approach is now formally recommended for all Cochrane qualitative evidence syntheses and is used widely within WHO and several other global and national guideline-producing agencies including the UK National Institute for Health and Care Excellence (NICE). With the rapid uptake of the approach, members of the GRADE-CERQual coordinating team sought to undertake an evaluation of how the method has been used and, in doing so, to develop fidelity and reporting criteria to assist review authors when applying GRADE-CERQual. Similar evaluations have been carried out for GRADE [25–33] but this is the first instance for GRADE-CERQual. As qualitative evidence synthesis expands, methodological evaluations are emerging and increasingly needed, including evaluations of reporting standards [34, 35], of the use of theories and frameworks [36, 37] and specific QES methods or components of methods [38–40]. An evaluation of the use of GRADE-CERQual is critical for further improving the application of the approach within reviews conducted for WHO, Cochrane and other global and national agencies.

The objective of this evaluation was twofold. First, to describe the uptake to date of GRADE-CERQual by authors of QES—for example, how often, in what types of reviews, and in which fields of study. Second, to assess how review authors report their GRADE-CERQual assessments and to assess fidelity to the most recent guidance for applying the GRADE-CERQual published in 2018 in the journal *Implementation Science* (herewith referred to as “the guidance”). We intended to highlight good examples of use, identify common fidelity and reporting concerns, assist review authors to avoid these in the future, and identify where further methodological research, training and guidance is needed.

## Methods

This evaluation was conceived as a citation analysis and methods overview. First, it involved identification and descriptive analysis of all citations of GRADE-CERQual. Second, all syntheses that applied the four component version of GRADE-CERQual underwent a detailed examination of their conduct and reporting, and the extent to which fidelity to the published guidance was demonstrated. Methodologically, this evaluation fits within the recent evaluative 'tradition' for assessing reporting guidelines, most notably the PRISMA statement [41]. For example, Page and Moher [42], the latter being one of the founders of the reporting guidelines movement, identified over 100 'evaluations of the uptake and impact' of the PRISMA statement and its various extensions. In evaluating 'uptake' they specifically point to the role of citations. We acknowledge however that the GRADE-CERQual approach extends beyond reporting guidance by specifying procedures for conduct of the grading of qualitative findings. Nevertheless, we found citation analysis was the best available approach for evaluating uptake given that GRADE-CERQual can be used in non-published reviews, can be used but not cited, or can be cited but not used.

Our working definition of fidelity is: the extent to which use of GRADE-CERQual demonstrates integrity to the procedures for conduct and reporting as specified in the corpus of GRADE-CERQual methodological papers [10, 14–19]. While we opted for the term fidelity, Page & Moher[42] preferred the term 'adherence' which they defined as “the extent to which SRs [systematic reviews] comply with each item in the [PRISMA] statement” (p.4). Our choice of fidelity over adherence reflects the fact that while PRISMA carries the status of an almost-mandatory requirement, GRADE-CERQual is largely a value-added procedure.

It was especially important for the team to be reflexive in this evaluation as it was conceptualised and led by members of the GRADE-CERQual coordinating team [43]. The team was therefore well qualified to judge fidelity but limited by prior researcher allegiance [44, 45] to the approach. This was balanced by: using a coordinating team member (MW) and non-coordinating team members (RIZ, WC) to conduct the fidelity and reporting assessments; ensuring that evaluation team members did not evaluate reviews they co-authored to avoid potential bias (or spin) [44]; and using the published guidance papers as the benchmark. Also, we acknowledge that GRADE-CERQual requires ongoing development and improvement and therefore approached the evaluation with a genuine openness to understanding other review authors’ interpretation and application of the guidance.

### Publication search

Our aim was to identify all publications that had mentioned GRADE-CERQual or referenced a GRADE-CERQual methods paper. First, we searched for “CERQual” and “GRADE-CERQual” in titles and abstracts in MEDLINE, EMBASE, Scopus, Web of Science, CINAHL and in full-texts in Google Scholar. Search strategies and results are available in Additional file 1. All searches were conducted in August 2020. For papers not using either abbreviation we searched using the following combination of terms: confidence AND evidence AND reviews AND "Qualitative research" (in recognition that "in" and "from" are stop words in most databases). We then conducted forward citation searches for 18 methodological papers (see Additional file 2), including official guidance and descriptions of the GRADE-CERQual method, and known translations, in Google Scholar (using the Publish or Perish tool), Scopus and Web of Science. We deliberated the value of forward citation searches for known published reviews that used GRADE-CERQual but decided that these references would likely duplicate those retrieved by the full-text phrase searches on Google Scholar. No language, date, or geographical filters were applied. Where possible, MEDLINE duplicates were excluded at the database search stage.

### Part 1—citation analysis and descriptive overview

#### Stage 1.1: coding and charting: titles and abstracts

We imported details of authors, year, title, journal, volume, issues, pages, DOI, abstract and URL for all retrieved papers from Endnote into Excel and divided the citations between the co-authors. Co-authors independently coded their assigned references in Excel for language, type of publication and whether a GRADE-CERQual coordinating team member was a co-author (see Additional file 3 for coding questions and answers). Twenty percent of articles assigned to types of publication other than “review/synthesis” were independently co-coded by a second researcher to assess whether unlabelled syntheses/reviews had eluded full-text coding/charting. As a team, we decided to exclude theses and dissertations to focus on comparable units of reporting detail from within the peer-reviewed literature. Spanish, Portuguese, French, Korean, Mandarin, Swedish, and Norwegian publications were coded by authors proficient in each language. We used Google Translate to translate abstracts and titles in other languages.

#### Stage 1.2: coding and charting: full texts

We uploaded publications coded to “Review/Synthesis (quantitative or qualitative)” at the title/abstract stage to the Covidence online screening platform [46]. We prepared an extraction form for full-text coding including guidance cited, field of study, whether GRADE-CERQual was applied, and type of review (Additional file 3). Each publication was coded by one author. Where the team had previously misattributed a publication as a review or synthesis when it was a protocol, a dissertation, a methodological, or conceptual paper, etc., then it was returned to the title/abstract coding stage. All such publications were then reviewed by the first author who verified the misattribution and updated the title/abstract coding and charting spreadsheet. We were lenient when interpreting “Was GRADE-CERQual applied to review findings?”; we selected “yes” if authors simply stated that they had used or applied GRADE-CERQual, even when the application was unclear. Co-authors proficient in that language coded all publications in languages other than English and 20% of English language publications were checked by a second author. MW and RIZ, who had coded most of the publications, also checked all references coded by other authors to further improve consistency.

### Part 2: fidelity and reporting assessment and analysis

#### Stage 2.1: coding for serious fidelity issues

When planning the evaluation, we anticipated that we would flag reviews that applied GRADE-CERQual to quantitative systematic reviews as having serious fidelity issues and that these would therefore not proceed to full reporting and fidelity assessment. However, close reading of each study revealed a need to flag other serious deficiencies (“fatal flaws”) in fidelity to the approach. Our list of fatal flaws is shown in Table 1. Only studies that applied GRADE-CERQual to assess confidence in individual review findings and were supported exclusively by findings from qualitative research proceeded to full reporting and fidelity assessment. At this stage we also coded for review type, synthesis method, and co-authorship or acknowledgement of members of the GRADE-CERQual coordinating team. In addition, we flagged studies thought to demonstrate an innovative use of the GRADE-CERQual approach.

**Table 1:** Questions and responses for identifying fatal flaws

Questions	Responses
How was GRADE-CERQual used?	CORRECT 1. Applied to assess confidence in individual review findings
	FATAL FLAWS 2. Applied as an assessment of general confidence in review findings, or the review itself as a whole 3. Applied as a critical appraisal tool for included studies 4. Applied as a critical appraisal or reporting tool for the review
What type of data were underlying the review findings that GRADE-CERQual was applied to?	CORRECT 1. Qualitative
	FATAL FLAWS 2. Qualitative and Quantitative 3. Quantitative only

#### Stage 2.2: reporting and fidelity assessment

We developed and finalised the reporting and fidelity assessment criteria (Additional file 4) through multiple rounds of pilot testing. First, we developed an expanded version of previously published minimum criteria for fidelity to the GRADE-CERQual approach [14]. All co-authors then piloted this version on two publications. Second, we discussed our experiences and feedback, and decided to separate assessment into ‘reporting’ and ‘fidelity’. Third, the first author redrafted the criteria and these were then piloted by some co-authors on a third article. Finally, we compared and discussed our assessments to confirm further modifications.

MW and RIZ built an NVivo12 [47] master project in which the reporting and fidelity assessment criteria would be applied. Each publication was added to both an overall case node (“evaluated studies”) and to a case node specific to the publication. We created a case classification sheet to register responses to each reporting and fidelity question. We also created a node for each criterion and data extracts were coded to these nodes to justify concerns with fidelity or reporting. We then created a framework matrix in NVivo12 to summarise concerns regarding fidelity. MW and RZ conducted simultaneous real-time pilot-testing, followed by minor revisions of the NVivo12 project structure against a single paper. MW and RIZ then independently pilot-tested the framework on two publications and met to compare assessments. Very minor revisions were made to improve the clarity of the responses to each question and a further two papers were independently assessed. MW and RIZ subsequently divided the remaining studies between them for single author assessment.

For analysis, RIZ looked for patterns in the data by exporting the case classification sheet to Excel and running counts of the data. MW applied a conventional qualitative content analysis approach [48] to sub-coding all the data illustrating concerns for each reporting and fidelity criteria. Qualitative content analysis was considered appropriate for descriptively and inductively identifying categories of concerns for each reporting and fidelity criteria and for observing their prevalence. In presenting our results we have deliberately avoided ‘naming and shaming’ individual studies. Instead, we seek to offer constructive feedback, to help review authors improve their reporting and fidelity and to identify how the GRADE-CERQual project group can further improve guidance for review authors.

## Results

**Fig. 1:**
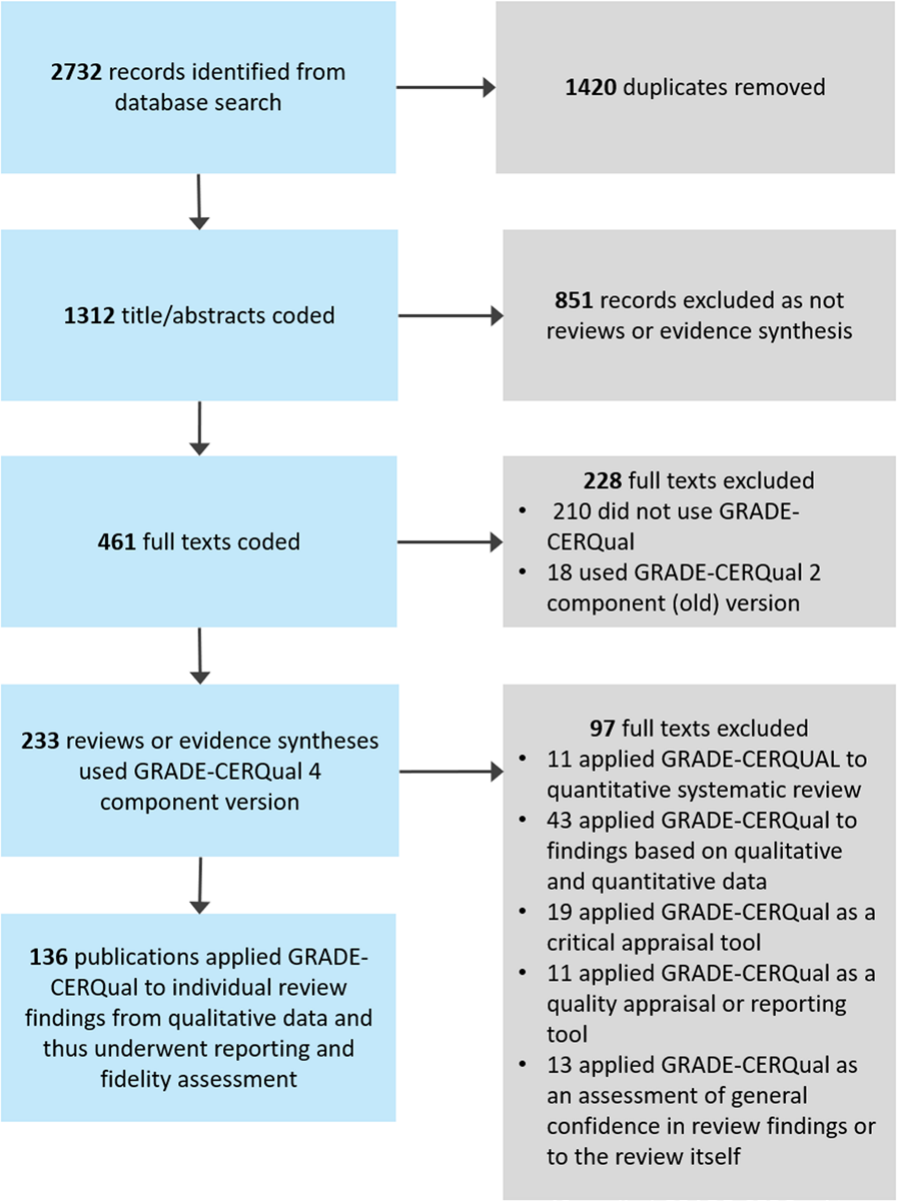
PRISMA flowchart

**Fig. 2:**
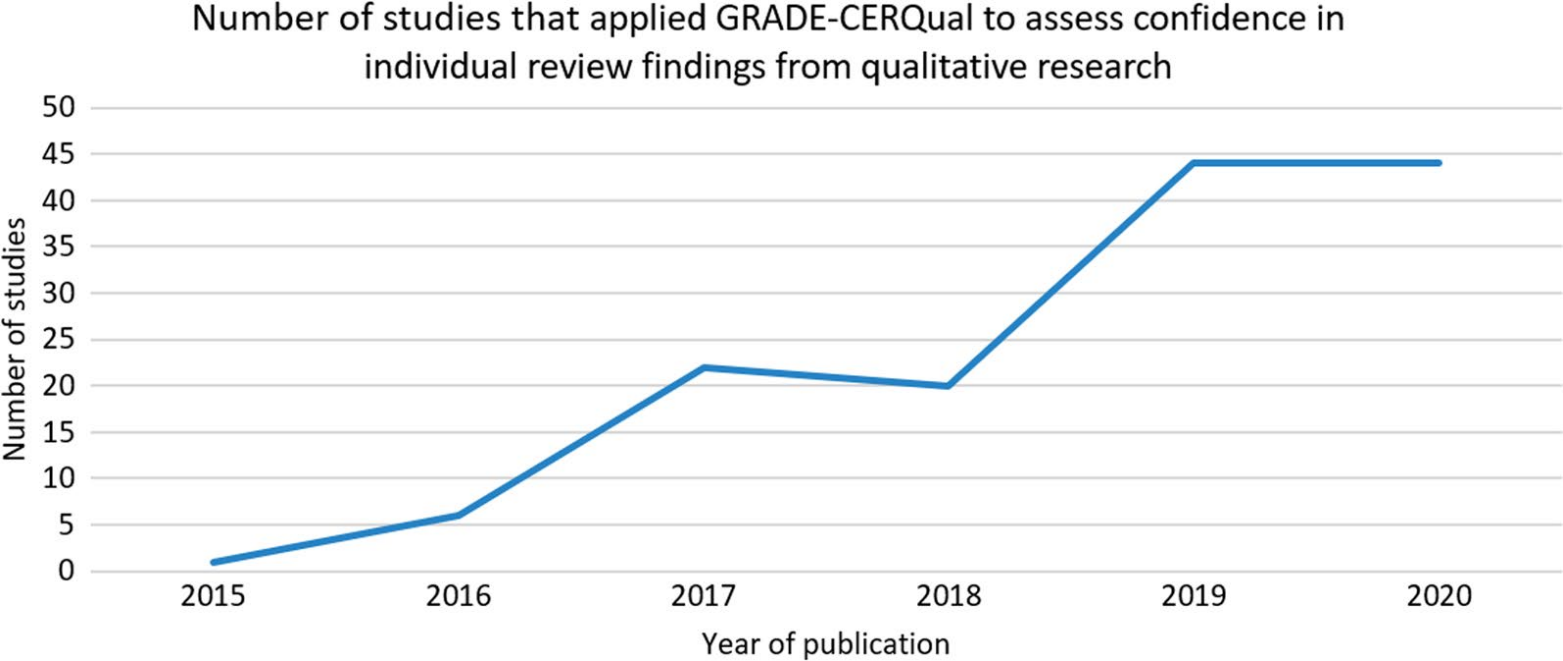
Number of published studies that applied GRADE-CERQual to individual review findings by year

A total of 2732 publications were found from database searches. After duplicates were removed, 1312 publications were assessed at the title/abstract stage. We identified 461 publications as reviews or evidence synthesis at the full text stage, of which 233 (50.5%) publications applied the four component version of GRADE-CERQual. Out of these 233 publications, a total of 136 publications (58.4%) applied GRADE-CERQual to individual review findings and were assessed for fidelity and reporting. Figure 1 shows the number of publications identified and included across the different stages of the evaluation. Overall, the number of published studies that applied GRADE-CERQual to individual review findings is increasing (Fig. 2).

### Part 1. Citation analysis and descriptive overview

There were 462 records coded as a review or synthesis (either quantitative, qualitative or mixed-methods) and proceeded to full-text coding and charting. Quantitative reviews were included at this stage in recognition that GRADE-CERQual is occasionally wrongly applied to findings from quantitative effect reviews, and because we wanted to ensure that we captured this issue in the evaluation. Of the 461 reviews or syntheses, 251 (54.4%) actually applied GRADE-CERQual in their reviews, of which 233 publications (92.8% of those applying the approach) used the four component version, and 18 (7.2%) publications used the earlier two component version. All results of the title and abstract coding are available in Additional file 5. As reviews that used the four component version of GRADE-CERQual are the most relevant to this evaluation, we only present results for these 233 studies.

Most of these 233 studies used standard review or synthesis methods for synthesising primary qualitative research (*n* = 223, 95.7%), while seven (3%) used overview or umbrella review approaches and three (1.2%) used scoping review approaches. Currently there is no official guidance for applying GRADE-CERQual to overview or umbrella reviews or scoping reviews. We included these in our evaluation as an opportunity to learn how review authors adapted the guidance. Although 184 studies (78.9%) were published from 2018 onwards, the *PLOS Medicine* methods paper [9] remained the most commonly-cited guidance (*n* = 120, 51.5%). Twenty-six publications (11.1%) neither cited methodological guidance publications [9, 10, 14, 15, 15–19], nor cited a published review or other publication about GRADE-CERQual. Almost all (*n* = 223, 95.7%) of the publications were written in the English language, with the remainder in Mandarin (*n* = 5, 2.1%), Spanish (*n* = 3, 1.2%), Norwegian (*n* = 1, 0.4%), and Swedish (*n* = 1, 0.4%). See Additional file 6 for full results of full-text coding and charting.

### Part 2. Fidelity and reporting assessment and analysis

#### Serious misapplications of GRADE-CERQual

A total of 233 studies applied the four component version of GRADE-CERQual. We categorised 97 (41.6%) as having seriously misused GRADE-CERQual. Fatal flaws, to borrow a term used in critical appraisal, include fundamentally misinterpreting GRADE-CERQual as either: a quality appraisal tool of included studies (*n* = 19, 19.6%), a critical appraisal tool or reporting tool for the review itself (*n* = 11, 11.3%), or an approach to assess general confidence in all findings or in the review as a whole (rather than individual review findings) (*n* = 13, 13.4%). A very serious fatal flaw was that 11 studies (11.3%) wrongly applied GRADE-CERQual to findings from a quantitative systematic review. The last and most common fatal flaw (*n* = 43, 44.3%) was applying GRADE-CERQual to review findings developed from both qualitative and quantitative data. GRADE-CERQual is designed to be applied to review findings supported exclusively by qualitative data (that is data emanating from qualitative data collection methods and qualitative methods of analysis). Therefore, application of GRADE-CERQual to review findings supported by both qualitative and quantitative data (including descriptive quantitative studies like surveys), does not align with current guidance. A mixed-methods study or mixed-methods review can apply GRADE-CERQual to the findings of a qualitative evidence synthesis that has been conducted in parallel to a quantitative review. Guidance from the GRADE-CERQual project group does not yet exist for applying GRADE-CERQual to review findings supported by mixed evidence within convergent synthesis designs. Most of the 43 studies with this fatal flaw neither acknowledged that this application deviated from the guidance nor did they sufficiently explain how they adapted the assessment of each component to the quantitative origins of the data. It was challenging to identify that review findings were supported by qualitative and quantitative data as reporting gaps were common. For example, many did not clearly label that they were mixed-methods or mixed studies reviews and did not specify the design used for their synthesis (e.g., convergent, parallel). Although the current version of GRADE-CERQual has not been designed for use in a scoping review or an overview/umbrella review we included those types of reviews in this evaluation as an opportunity to learn how review authors had adapted the guidance. However, all three scoping reviews and six of the eight overview/umbrella reviews were excluded for one of the above mentioned fatal flaws. Unsurprisingly, the two umbrella reviews that went on to full assessment scored poorly on fidelity to the guidance.

#### Most common reporting issues

**Fig. 3:**
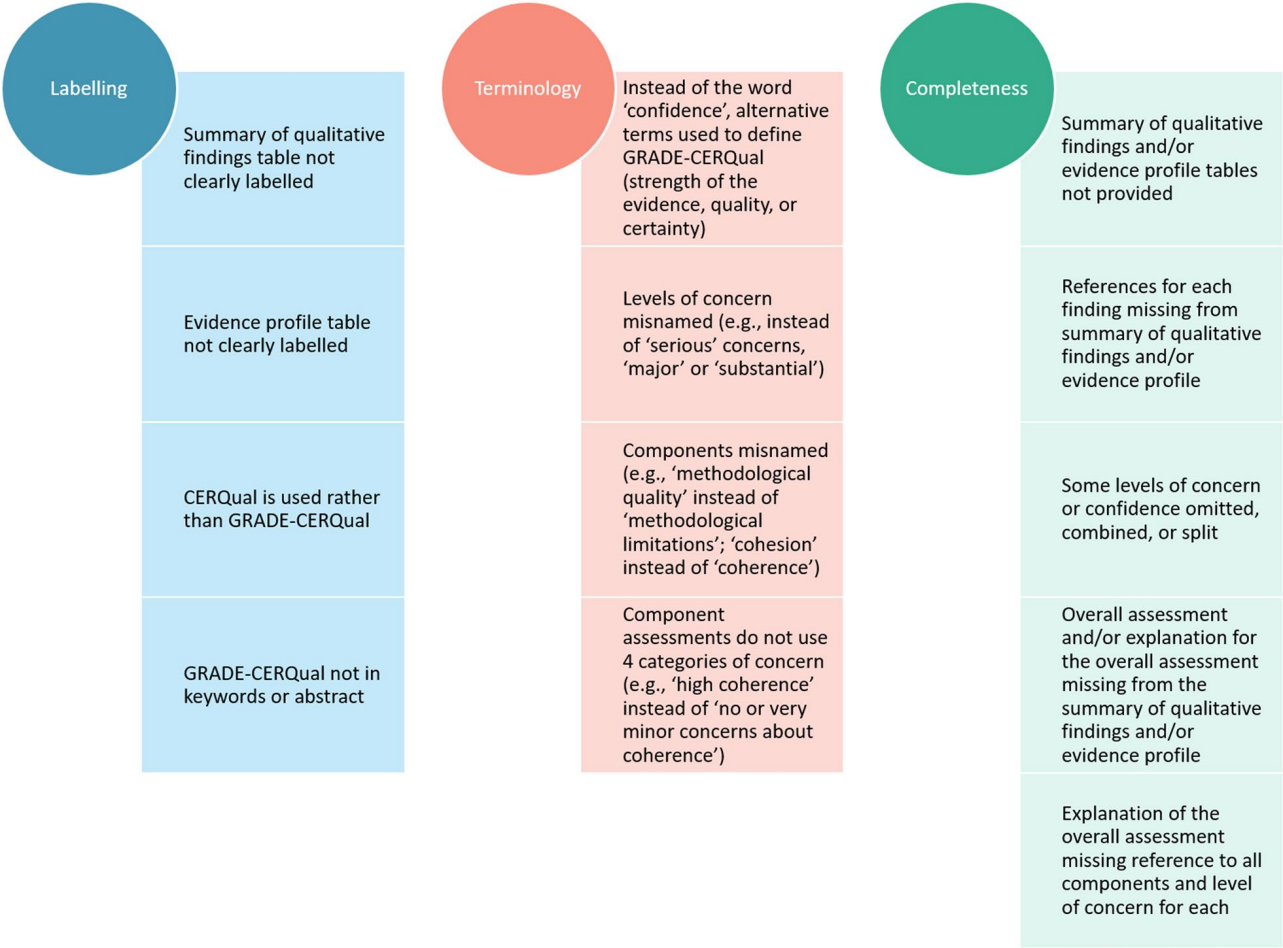
Main reporting concerns grouped by broad area

A total of 136 studies applied the four-component version of GRADE-CERQual to assess confidence in individual review findings and were consequently brought to the next stage of analysis. Full results of the reporting assessment of these 136 studies are available in Additional file 7. We synthesised the reporting issues into three broad areas: labelling, terminology, and completeness. Figure 3 summarises the main reporting concerns. The most common reporting issues relate to two key outputs; the Summary of Qualitative Findings (SoQF) Table and the Evidence Profile table. Though most reviews included one or other of these, they were often poorly labelled and omitted crucial elements such as an explanation for the overall assessment and the citations for the studies contributing to each summarised review finding.

#### Most common fidelity issues

**Fig. 4:**
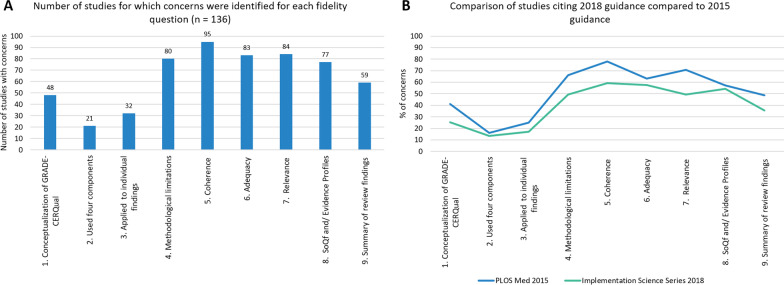
Graph showing concerns per fidelity criteria^.^ **a** Bar graph showing number of studies with concerns on each fidelity question; **b** Line graph comparing percentage of concerns between studies that cited 2015 and 2018 guidance for the GRADE-CERQual approach

As seen on Fig. 4a fidelity concerns were identified in over half of all studies assessed. Publications citing the 2018 *Implementation Science* guidance [14–19] were less likely to generate concerns regarding each fidelity criteria compared to publications citing the 2015 guidance [9] (Fig. 4b). It is important to note that in this evaluation we assessed all studies against the 2018 guidance. The largest improvements were seen in the greater clarity with which the GRADE-CERQual approach and each of the four components were conceptualised. This was to be expected given that the 2018 series published four separate papers detailing how to operationalise each component (see section “Steps” in each of these publications [16–19]). Mixed methods reviews tended to occasion more concerns, on average, than qualitative evidence syntheses.

Although the frequencies on Fig. 4 help to visualise patterns, they do encompass very minor through to serious concerns. In other words, any difference from the guidance was flagged as a concern, whether very minor or not. To address this, the qualitative content analysis sub-coding revealed the full range of concerns for each criteria (full results in Additional file 8). Table 2 is an abbreviated version highlighting the most common, alongside what we considered to be the most important, concerns. We found that these identified concerns often reflected wider conceptual or operational concerns in the review as a whole. In the following sections we discuss and reflect on these cross-cutting issues.

**Table 2:** Common concerns identified on fidelity assessment

Fidelity assessment questions Common concerns identified in bullet points
*1. The authors demonstrate an accurate conceptualisation of GRADE-CERQual (that is, an approach for assessing confidence in the findings of a qualitative evidence synthesis)* • Appears under quality appraisal section • Sometimes referred to as a tool to assess quality of findings or evidence • Conceptualised as an assessment of contributing studies
*2.The authors have made an overall assessment of confidence based on the assessment of all four components* • No mention of the 4 components at all • Some components not assessed • Applied own scoring rules for determining level of assessment
*3.The authors applied GRADE-CERQual to individual review findings* • Applied GRADE-CERQual at the study level not finding level • Applied it to short theme or category titles
*4.Authors conceptualise methodological limitations in line with the guidance* • Applied the levels of concern to individual studies rather than review findings • Conceptualised the assessment as a count of appraisal categories, not specific limitations in relation to the finding • Component not defined and no Evidence Profile or SoQF tables from which to infer • Not conceptualised in terms of identifying concerns • Problems with how critical appraisals were done (e.g., only yes or no, no explanation) • Specific methodological limitations mentioned but not how important they are in relation to the finding
*5. Authors conceptualise coherence in line with the guidance* • Component not defined and no Evidence Profile or SoQF tables from which to infer • No demonstration of thinking of it in terms of the fit between review finding and data from primary studies, only focus on primary studies • Not conceptualised in terms of identifying concerns • Using wrong definition (“Consistent within and across studies”) • Assessment was quantified
*6. Authors conceptualise adequacy of data in line with the guidance* • Component not defined and no Evidence Profile or SoQF table from which to infer • Not assessed in terms of concerns • Not assessing both quantity and richness, emphasising one or the other • Confounding with other components • Quantify the assessment of the component
*7. Authors conceptualise relevance in line with the guidance* • Component not defined and no Evidence Profile or SoQF tables from which to infer • Language of concerns not used, or not used correctly • Not all elements of ‘context’ were considered in the assessment • Quantify the assessment by counting how many primary studies are indirect or partial, rather than identifying concerns
*8.The GRADE-CERQual assessments are presented in-line with the guidance for SoQF tables and or Evidence Profiles* • No SoQF or Evidence Profile tables included • Key elements missing or left out (such as references or explanations) • Way of writing explanations for component or overall assessments not aligned with guidance
*9. Summarised review findings were produced in line with the guidance* • Summaries of findings either too detailed or too brief • Just theme or category names, not summarised review findings

Of the 136 studies we assessed for fidelity, the most common synthesis methods used were thematic synthesis (*n* = 69, 50.7%), followed by meta-ethnography (*n* = 18, 13.2%). The GRADE-CERQual coordinating team has long acknowledged that experience to date has focused on applying GRADE-CERQual to descriptive findings with a need for more application to interpretive or analytic findings. It is encouraging to see many meta-ethnographies emerging that apply GRADE-CERQual. However, fidelity varied widely between meta-ethnographies, demonstrating a need for clearer guidance on applying GRADE-CERQual to these types of syntheses. While we did not identify any examples with a complete absence of concerns, four meta-ethnographies had no concerns for most criteria [49–52].

#### Examples of good fidelity

We did not identify any concerns for eleven publications. Six of these were Cochrane Reviews [53–58] and two were reports of commissioned reviews [59, 60] where review authors are given sufficient word limits to fully describe the GRADE-CERQual approach and demonstrate how it was applied. In comparison, journal articles may have more restrictive word limits. All of the Cochrane reviews involved GRADE-CERQual coordinating team members as either co-authors or acknowledged mentors. The remaining four publications were published in academic journals [61–64]^.^and all included additional files. As writing within word limits is a serious challenge when publishing systematic reviews, including details of GRADE-CERQual assessments (such as Evidence Profile or SoQF tables) in additional files is crucial for demonstrating fidelity to the approach and transparency in one's judgements. Since conceptual and operational fidelity to the guidance for assessing each of the four components needs to be improved, we recommend the following six studies as good examples. Collectively, these studies included an Evidence Profile table for their GRADE-CERQual assessments and did not raise any concerns for how they conceptualised the components [52, 65–69].

## Discussion

Our evaluation identified several broad issues which can better inform users of the GRADE-CERQual approach, including guideline developers, review authors and researchers. These issues include conceptual challenges, problems with the application of the approach, and inadequate reporting that compromises transparency. The following discussion considers each in turn.

### Conceptual challenges related to applying GRADE-CERQual

#### Confusion between confidence in review findings and quality

GRADE-CERQual is an approach for assessing confidence in, rather than the quality of, review findings. In our evaluation, we found several indicators of conceptual confusion between ‘quality’ and ‘confidence’. These included the words being used interchangeably; review authors describing the GRADE-CERQual approach in the section of the article addressing quality appraisal of primary studies; and suggestions that GRADE-CERQual was intended to assess the quality of review findings. However, in the context of qualitative evidence syntheses, the "quality" of a review finding is not a useful concept—it is more appropriate to consider how much confidence we have in a review finding. Further, while assessing the methodological limitations of studies included in a synthesis (sometimes called ‘quality appraisal’) is a key stage of the review process, this should not be confused with assessing confidence in a review finding. A review finding is typically based on data from several studies, each with their own methodological limitations. As we have described elsewhere [16], the methodological limitations component of GRADE-CERQual requires that review authors have previously applied a critical appraisal tool to all contributing studies, and then consider the importance of these methodological limitations in relation to each review finding.

#### Poor understanding of the kind of review GRADE-CERQual can and cannot be applied to

Despite GRADE-CERQual being an approach for use in qualitative evidence syntheses, eleven reviews of quantitative evidence chose to use GRADE-CERQual. This was typically based on the misinterpretation that GRADE-CERQual can be applied to narrative summaries of quantitative results (i.e. a synthesis without meta-analysis). A synthesis without meta-analysis is not a qualitative evidence synthesis and should not be described as ‘qualitative’ as this contributes to confusion. In fact, recent guidance for reporting quantitative synthesis without meta-analysis also deliberately avoids the term narrative synthesis [70]. The bottom line is GRADE-CERQual has been designed for assessment of findings grounded in data from primary studies that used qualitative methods for collecting and analysing data. GRADE for effectiveness is the appropriate approach for review findings based on quantitative data, even in cases where a meta-analysis was not possible [71]. Numerous approaches are emerging to guide users in selecting an analysis approach appropriate to the aim and underlying data of their systematic review [70, 72]. Furthermore, considering that the most common fatal flaw was applying GRADE-CERQual to review findings developed from both qualitative and quantitative data (e.g. survey data), points to a gap in available methods for assessing confidence in findings from mixed methods or mixed studies reviews using convergent designs (where quantitative data is transformed into qualitative form and analysed and synthesised together with qualitative data).

### Problems with the application of the approach

#### Specific review findings versus overall themes

GRADE-CERQual is applied to individual review findings. In some reviews, these findings may be organised into broader theme headings but, in these cases, GRADE-CERQual should still be applied to the granular review findings. For example, “inequality in access to information” could be a theme identified in a synthesis, and this theme may include specific review findings—for instance, “Unlike women under 40, women over 40 faced technological barriers to accessing the information they needed on their diagnosis”. In this case GRADE-CERQual should be applied to the specific review finding(s) and not the theme category. However, the authors of 14 reviews appeared to apply GRADE-CERQual to overall themes and subthemes, instead of the summaries of specific review findings. Instead of presenting a summary of a review finding in the SoQF or Evidence Profile, only a brief theme label (no more than 3–5 words) was provided. This is not congruent with the guidance because the summary of the finding needs to provide sufficient detail to be understood by readers of the tables. One way to demonstrate in an SoQF or Evidence Profile that some review findings fall within the same theme is to divide these tables into sections corresponding to these theme headings [73], or to include the brief theme label alongside a summary of the relevant findings [74].

#### Quantifying GRADE-CERQual assessments

The quantification of GRADE-CERQual assessments proved to be one of the most concerning fidelity issues. In the most extreme form of quantification, Evidence Profile tables only contained numbers of studies without explanations. For example, a review might state how many studies achieved each quality rating for methodological limitations, and how many indirectly or partially relevant studies contributed to relevance. While reference to the quantity of studies may help to explain an assessment, that assessment itself is not a count. The assessment for each component is a judgement of appropriate concerns regarding that component. Similarly, the overall assessment of confidence as either high, moderate, low or very low is a judgement, not a count. It was concerning that some authors determined confidence to be “high” based on whether the finding was supported by a given number of studies or cases. This deviation from the guidance completely overlooks the need to factor-in concerns noted for all four components when making the overall assessment. Some authors also deviated from the guidance by establishing scoring systems to determine overall confidence – e.g., low confidence for one component with serious concerns, or two components with moderate concerns. Scoring was considered a concern rather than an innovation on the GRADE-CERQual approach because of over-reliance on quantification and the false sense of precision that this provides [15].

#### Assessing components at the individual study rather than the review finding level

The error of quantifying assessments is also tied closely to a conceptual issue around assessing the GRADE-CERQual components at the individual study level rather than the review finding level. GRADE-CERQual is an assessment of confidence in the review finding. Thus, while component assessment involves looking back at the included studies supporting the finding, the review finding is being assessed, not the contributing studies. The categories of no or very minor, minor, moderate, or serious concerns are applied to the review finding, not to individual supporting studies. Some review authors applied the components to the individual study, assigning each contributing study a level of concern for each component, and then reporting a count of how many supporting studies registered each level of concern. The focus is deflected towards the sheer number of concerns aggregated from primary studies, and away from a careful consideration of the importance that review authors assign to these concerns in relation to the specific review finding that they are assessing.

#### GRADE-CERQual components not assessed in terms of concerns

A further issue relates to authors not conceptualising component assessments in terms of concerns at all, as was the case in 36 reviews. Some authors confused the assessment categories for the overall assessment of confidence with the assessment categories for component assessments. For example, instead of indicating no or very minor concerns about relevance, authors might assign high or moderate relevance. This departs from the guidance, given “our aim is not to judge whether data [component] has been achieved, but to judge whether there are grounds for concern regarding [the component] that are serious enough to lower our confidence in the review finding” [18]. In other instances, authors just used the component name as the assessment – e.g., “adequate data” rather than “no/very minor concerns for adequacy of data”. This again points to a subtle but nonetheless significant tendency to frame component assessments in terms of whether an ideal has been met, rather than identifying grounds for concern.

#### Issues specific to each component

The evaluation identified several issues specific to each GRADE-CERQual component (methodological limitations, coherence, relevance, and adequacy of data). The most common fidelity concern related to coherence being wrongly defined as “consistency within and across studies”. Other frequent concerns related to reporting of methodological limitations and how such limitations were considered in relation to each review finding. Considerations of relevance and adequacy focused on a limited interpretation of these components (e.g., for adequacy focusing on either quantity or richness but not both, and for relevance focusing only on one aspect like setting (thus overlooking other aspects of context like perspective, population or phenomenon of interest). Some review authors utilised concepts related to one component to assess another. These component-specific issues are described in detail in Additional file 9.

### Reporting that compromises transparency

Inadequate reporting compromises transparency. GRADE-CERQual assessments are judgements made by review authors, therefore, transparency is key to understanding these judgements and is therefore fundamental to the overall approach. To demonstrate adherence to the principle of transparency, review authors must provide an explanation for each of their component assessments and their overall assessment of confidence. Summarising assessments for each component within an explanation for the overall assessment cannot replace individual explanations for each component. Furthermore, an overall assessment is incomplete without an explanation. We recommend that, as a minimum, the explanation for the overall assessment should state the level of concern for each component. However, we encourage authors to add additional detail about the concerns that are driving down their confidence in the finding. An important part of transparency is including an SoQF table and Evidence Profile table. Assessing fidelity based only on a SoQF table is very difficult given insufficient detail for the reader to understand how review authors interpreted and assessed each component. We therefore recommend that qualitative evidence syntheses include Evidence Profiles as additional files.

## Strengths and limitations

Here we reflect on the strengths and limitations, firstly, of the evaluation, and, secondly, of the GRADE-CERQual approach. A strength of this evaluation is that we systematically assessed each included review against specific criteria, and have clearly identified fatal flaws and most common reporting and fidelity issues, with the aim of being as useful as possible to future review authors. We consider this evaluation an essential complement to our published methodological guidance. Limitations of our evaluation include being reliant only on published materials (we did not have the resources to contact review authors for additional information), and having potentially missed relevant applications of GRADE-CERQual in our pragmatic decision to exclude theses and dissertations. Possible issues related to applying the approach in the context of a degree-related output were not identified.

This evaluation shows that an important strength of the GRADE-CERQual approach is that review authors have found GRADE-CERQual to be applicable to a wide range of topics and to different types of qualitative evidence synthesis. Limitations of the approach include that it has not yet been adapted for mixed methods reviews with convergent designs. The current version of GRADE-CERQual is not intended to be applied to such review findings but there is no alternative approach for review authors to use. This is an important area for the future development of the GRADE-CERQual approach. Furthermore, review authors could benefit from summaries of the published guidance, focusing in particular on the “steps” for assessment section of the papers. The new GRADE-CERQual iSoQ tool (isoq.epistemonikos.org) is expected to assist review authors with following the steps in the approach.

## Conclusions—future agendas

This study identified the most common and serious reporting and fidelity concerns when applying the GRADE-CERQual approach. The fidelity and reporting criteria used for the evaluation can help review authors to avoid common pitfalls and improve alignment with the guidance. This evaluation has also identified key areas for future research (Table 3), and future priorities for the dissemination and implementation of the GRADE-CERQual approach (Table 4). Future research could evaluate uptake and use beyond review authors, to include for example the use of GRADE-CERQual assessments by decision makers.

**Table 3:** Future research agenda for the GRADE-CERQual approach

• Refine the criteria that need to be met for authors to claim fidelity to the GRADE-CERQual approach, drawing on the assessment criteria used in this evaluation
• Develop reporting criteria for the use of GRADE-CERQual in qualitative evidence syntheses, drawing on the reporting assessment criteria used in this evaluation
• Clarify guidance on the types of analytic outputs, or findings, from a qualitative evidence synthesis to which GRADE-CERQual should be applied, including in the context of a meta-ethnography
• Improve the guidance on applying each GRADE-CERQual component, focusing on: (1) helping authors to conceptually navigate the boundary between considering concerns at the individual study level and assessing the importance of these concerns at the review finding level; (2) navigating the conceptual boundaries between components
• Develop more detailed guidance on how to bring together GRADE-CERQual component assessments to make an overall assessment of confidence, while avoiding quantification of individual component assessments
• Evaluate the impact of the new GRADE-CERQual interactive Summary of Qualitative Findings (iSoQ) tool on the completeness and consistency of reporting of GRADE-CERQual assessments, including explanations of these, and on the presentation of Summary of Qualitative Findings and Evidence Profile tables

**Table 4:** Future dissemination and implementation agenda for the GRADE-CERQual approach

• Improve the accessibility of guidance on component assessments and making an overall assessment, for example by disseminating concise instructions for these
• Encourage review authors, and journals publishing qualitative evidence syntheses, to include SoQF tables in all qualitative evidence syntheses and to make GRADE-CERQual Evidence Profiles available as supplementary materials
• Explore how good examples identified in this evaluation of the application of GRADE-CERQual can be used to support training and other forms of capacity strengthening for applying the approach
• Continue to identify examples of GRADE-CERQual applications that adhere to our current reporting and fidelity criteria so that these can be used for capacity strengthening
• Explore how training and guidance materials linked to the new GRADE-CERQual interactive Summary of Qualitative Findings (iSoQ) tool could be used to strengthen capacity to apply the GRADE-CERQual approach appropriately
• Encourage the incorporation of training and guidance materials on the GRADE-CERQual approach into teaching modules on qualitative evidence synthesis and on systematic reviewing more broadly

## Supplementary Information

**Additional file 1. **Search log: GRADE-CERQual topic and citation searches

**Additional file 2.** List of GRADE-GRADE-CERQual publications for citation searching

**Additional file 3.** Coding and charting questions and answers

**Additional file 4. **Reporting and fidelity assessment criteria

**Additional file 5. **Results of title and abstract coding and charting

**Additional file 6. **Results of full-text coding and charting that applied GRADE-CERQual

**Additional file 7. **GRADE-CERQual reporting assessment results

**Additional file 8.** GRADE-CERQual reporting and fidelity assessment sub-coding of identified concerns by criteria

**Additional file 9.** Issues specific to each component

## Data Availability

Additional information files are provided, and further data may be provided upon reasonable request.

## Abbreviations

GRADE-CERQual: Confidence in the Evidence from Reviews of Qualitative research
iSoQ: GRADE-CERQual interactive Summary of Qualitative Findings (iSoQ) tool
NICE: National Institute for Health and Care Excellence
QES: Qualitative evidence synthesis
SoQF Table: Summary of Qualitative Findings Table
