# Supplementary material for: The use of GRADE-CERQual in qualitative evidence synthesis: an evaluation of fidelity and reporting
Source: Health Res Policy Syst. 2023 Jul 25;21:77. doi: 10.1186/s12961-023-00999-3 (PMC10369711; doi:10.1186/s12961-023-00999-3)
Supplement: Supplementary file 1 — Additional file 1. Search log: GRADE-CERQual topic and citation searches [file 12961_2023_999_MOESM1_ESM.docx]

# **Additional file 1**

## **Search log: GRADE-CERQual topic and citation searches**

| Source | Date searched | Search strategy | Hits (or records obtained from searches) | Comments |
| --- | --- | --- | --- | --- |
| PubMed MEDLINE | 05/08/2020 | GRADE-CERQual OR “Grade GRADE-CERQual” OR (Confidence AND Evidence AND Reviews AND "Qualitative research") | 254 | GRADE-CERQual also retrieves “GRADE GRADE-CERQual” but both are included to protect against database search functionality variations |
| EMBASE | 05/08/2020 | 1 "grade GRADE-CERQual".mp. [mp=title, abstract, heading word, drug trade name, original title, device manufacturer, drug manufacturer, device trade name, keyword, floating subheading word, candidate term word] 82  2 (Confidence and Evidence and Reviews and "Qualitative research").mp. [mp=title, abstract, heading word, drug trade name, original title, device manufacturer, drug manufacturer, device trade name, keyword, floating subheading word, candidate term word] 108  3 GRADE-CERQual.mp. 136  4 1 or 2 or 3 181  5 limit 4 to medline 44  6 4 not 5 137 | 137 | (excludes MEDLINE – 181 if included) |
| Scopus | 05/08/2020 | TITLE-ABS-KEY ( ( GRADE-CERQual OR "Grade GRADE-CERQual" OR ( confidence AND evidence AND reviews AND "Qualitative research" ) ) ) 294 results | 294 | No functionality to Exclude MEDLINE |
| Web of Science | 05/08/2020 | TOPIC: (GRADE-CERQual OR “Grade GRADE-CERQual” OR (Confidence AND Evidence AND Reviews AND "Qualitative research")) 289 results | 35 | Excludes 254 MEDLINE (Above) |
| CINAHL | 05/08/2020 | TX GRADE-CERQual OR “Grade GRADE-CERQual” OR (Confidence AND Evidence AND Reviews AND "Qualitative research") | 132 | No functionality to Exclude MEDLINE |
| Citation Searches  Google Scholar | 17/08/2020 | [First 18 refs from Wainwright list] | 784 refs |  |
| Citation Searches  Web of Science | 17/08/2020 | [First 18 refs from Wainwright list] | 568 refs |  |
| Citation Searches Google Scholar | 18/08/2020 | Glenton Review (#19 from list) | 420 refs | Subsequently decided not to use this citation search (not methodological ref) |
| Citation Searches  Web of Science | 18/08/2020 | Glenton Review (#19 from list) | 223 refs | Subsequently decided not to use this citation search (not methodological ref) |
| Google Scholar (Publish or Perish Interface) | 21/08/2020 | GRADE-CERQual (Maximum 1000) | 1000 | Search stopped at 1000  2010-2020 |
| Google Scholar (Publish or Perish Interface) | 21/08/2020 | “Confidence in the Evidence from Reviews of Qualitative research” | 404 | 2010-2020 |
