# Supplementary material for: The use of GRADE-CERQual in qualitative evidence synthesis: an evaluation of fidelity and reporting
Source: Health Res Policy Syst. 2023 Jul 25;21:77. doi: 10.1186/s12961-023-00999-3 (PMC10369711; doi:10.1186/s12961-023-00999-3)
Supplement: Supplementary file 2 — Additional file 2. List of GRADE-GRADE-CERQual publications for citation searching [file 12961_2023_999_MOESM2_ESM.docx]

# **Additional file 2**

## **List of GRADE-GRADE-CERQual publications for citation searching**

1. Lewin S, Booth A, Glenton C, Munthe-Kaas H, Rashidian A, Wainwright M, Bohren MA, Tunçalp Ö, Colvin CJ, Garside R, Carlsen B, Langlois EV, Noyes J. **Applying GRADE-GRADE-CERQual to qualitative evidence synthesis findings: introduction to the series**. Implement Sci. 2018 Jan 25;13(Suppl 1):2
2. Lewin S, Bohren M, Rashidian A, Munthe-Kaas H, Glenton C, Colvin CJ, Garside R, Noyes J, Booth A, Tunçalp Ö, Wainwright M, Flottorp S, Tucker JD, Carlsen B. **Applying GRADE-GRADE-CERQual to qualitative evidence synthesis findings-paper 2: how to make an overall GRADE-CERQual assessment of confidence and create a Summary of Qualitative Findings table**. Implement Sci. 2018 Jan 25;13(Suppl 1):10.
3. Munthe-Kaas H, Bohren MA, Glenton C, Lewin S, Noyes J, Tunçalp Ö, Booth A, Garside R, Colvin CJ, Wainwright M, Rashidian A, Flottorp S, Carlsen B. **Applying GRADE-GRADE-CERQual to qualitative evidence synthesis findings-paper 3: how to assess methodological limitations**. Implement Sci. 2018 Jan 25;13(Suppl 1):9.
4. Colvin CJ, Garside R, Wainwright M, Munthe-Kaas H, Glenton C, Bohren MA, Carlsen B, Tunçalp Ö, Noyes J, Booth A, Rashidian A, Flottorp S, Lewin S. **Applying GRADE-GRADE-CERQual to qualitative evidence synthesis findings-paper 4: how to assess coherence**. Implement Sci. 2018 Jan 25;13(Suppl 1):13.
5. Glenton C, Carlsen B, Lewin S, Munthe-Kaas H, Colvin CJ, Tunçalp Ö, Bohren MA, Noyes J, Booth A, Garside R, Rashidian A, Flottorp S, Wainwright M. **Applying GRADE-GRADE-CERQual to qualitative evidence synthesis findings-paper 5: how to assess adequacy of data**. Implement Sci. 2018 Jan 25;13(Suppl 1):14.
6. Noyes J, Booth A, Lewin S, Carlsen B, Glenton C, Colvin CJ, Garside R, Bohren MA, Rashidian A, Wainwright M, Tun?alp Ö, Chandler J, Flottorp S, Pantoja T, Tucker JD, Munthe-Kaas H**. Applying GRADE-GRADE-CERQual to qualitative evidence synthesis findings-paper 6: how to assess relevance of the data**. Implement Sci. 2018 Jan 25;13(Suppl 1):4.
7. Booth A, Lewin S, Glenton C, Munthe-Kaas H, Toews I, Noyes J, Rashidian A, Berg RC, Nyakang'o B, Meerpohl JJ; GRADE-GRADE-CERQual Coordinating Team. **Applying GRADE-GRADE-CERQual to qualitative evidence synthesis findings-paper 7: understanding the potential impacts of dissemination bias**. Implement Sci. 2018 Jan 25;13(Suppl 1):12.
8. Toma, T.S., Barreto, J.O.M. **GRADE-GRADE-CERQual: uma abordagem para avaliar a confiança nos resultados de sínteses de evidências qualitativas.** São Paulo; Instituto de Saúde; 2019. 220 p p. ilus.(Temas em saúde coletiva, 27). Monografia em Português | Sec. Est. Saúde SP, SESSP-ISACERVO | ID: biblio-1025382 Biblioteca responsável: BR1764.1 Localização: BR1764.1; 613, G76. <https://pesquisa.bvsalud.org/portal/resource/pt/biblio-1025382>
9. Simon Lewin, Claire Glenton, Heather Munthe-Kaas, Benedicte Carlsen, Christopher J. Colvin, Metin Gülmezoglu, Jane Noyes, Andrew Booth, Ruth Garside, and Arash Rashidian. **Using qualitative evidence in decision making for health and social interventions: an approach to assess confidence in findings from qualitative evidence syntheses (GRADE-GRADE-CERQual)**. PLoS Med 12, no. 10 (2015) 001895.
10. **定性系统评价证据分级工具——GRADE-CERQual简介** [translation of PLoS Med 2015] Chinese Journal of Evidence Based Medicine. 2015, 15(12): 1465-14. <http://www.cjebm.com/article/10.7507/1672-2531.20150238>
11. Simon Lewin, Claire Glenton, Heather Munthe-Kaas, Benedicte Carlsen, Christopher J. Colvin, Metin Gülmezoglu, Jane Noyes, Andrew Booth, Ruth Garside, and Arash Rashidian. **Uso de Evidência Qualitativa em Decisões para Intervenções Sociais e em Saúde: Uma Abordagem para Avaliar a Confiança em Achados de Sínteses de Evidências Qualitativas (GRADE-GRADE-CERQual)** PLoS Med 12, no. 10 (2015) 001895.
12. Colvin, C., Wainwright, M., Noyes, J., Munthe-Kaas, H., Garside, R., Carlsen, B., Rashidian, A., Booth, A., Glenton, C., Bohren, M., Lewin, S., Tuncalp, O. **Confidence in the Evidence from Reviews of Qualitative research (GRADE-CERQual): Development and Future Directions of a Novel Approach**. In Chandler J, McKenzie J, Boutron I, Welch V (editors) Cochrane Methods. Cochrane DB Syst Rev 2015 Suppl 1: 45-47.
13. Langlois, E. V., Tunçalp, Ö., Norris, S. L., Askew, I., & Ghaffar, A. (2018). **Qualitative evidence to improve guidelines and health decision-making**. *Bulletin of the World Health Organization*, *96*(2), 79–79A. <https://doi.org/10.2471/BLT.17.206540>
14. **Using evidence from qualitative research to develop WHO guidelines**. In: WHO handbook for guideline development. 2nd ed. Geneva: World Health Organization; 2014.
15. **Uso de datos obtenidos mediante investigaciones cualitativas para elaborar las directrices de la OMS**. Manual para la elaboración de directrices- 2a edición. (ISBN 978 92 4 354896 8) © Organización Mundial de la Salud 2015 Traducción al español del capítulo 15 realizada por la Organización Panamericana de la Salud (OPS). [https://iris.paho.org/bitstream/handle/10665.2/28538/PAHOKBR16004_spa.pdf?sequence=1&isAllowed=y](https://eur01.safelinks.protection.outlook.com/?url=https%3A%2F%2Firis.paho.org%2Fbitstream%2Fhandle%2F10665.2%2F28538%2FPAHOKBR16004_spa.pdf%3Fsequence%3D1%26isAllowed%3Dy&data=02%7C01%7Cjane.noyes%40bangor.ac.uk%7C22520819b3ec4fdafd3708d839238b7b%7Cc6474c55a9234d2a9bd4ece37148dbb2%7C0%7C1%7C637322172136041991&sdata=%2BtoRdWpbcC6mDL%2FFECbhKz7UAhyKnMQsNr4W8jEgJcU%3D&reserved=0)
16. **Usando evidências de pesquisa qualitativa para desenvolver diretrizes da OMS**. Traduzido para o Português por Maria Sharmila Alina de Sousa e Ananyr Porto Fajardo a partir do Capítulo 15 Using evidence from qualitative research to develop WHO guidelines of the WHO handbook for guideline development, 2014 (ISBN 978 92 4 154896 0). [https://www.who.int/publications/guidelines/WHO_Handbook-for-Guideline-Develoment-Chapter-15_Portuguese.pdf?ua=1](https://eur01.safelinks.protection.outlook.com/?url=https%3A%2F%2Fwww.who.int%2Fpublications%2Fguidelines%2FWHO_Handbook-for-Guideline-Develoment-Chapter-15_Portuguese.pdf%3Fua%3D1&data=02%7C01%7Cjane.noyes%40bangor.ac.uk%7C22520819b3ec4fdafd3708d839238b7b%7Cc6474c55a9234d2a9bd4ece37148dbb2%7C0%7C1%7C637322172136041991&sdata=accmngyx3gMJiYCxy03QQo3KAvuq%2FQDXVluGHtDqtHI%3D&reserved=0)
17. Lewin S, Glenton C, Munthe-Kaas H, Carlsen B, Colvin C, Noyes J, Rashidian A. **Assessing how much certainty to place in findings from qualitative evidence syntheses: the GRADE-CERQual approach**. In: Better Knowledge for Better Health | Un meilleur savoir pour une meilleure santé. Abstracts of the 21st Cochrane Colloquium; 2013 19-23 Sep; Québec City, Canada. John Wiley & Sons; 2013. [https://abstracts.cochrane.org/2013-qu%C3%A9bec-city/assessing-how-much-certainty-place-findings-qualitative-evidence-syntheses-GRADE-CERQual](https://abstracts.cochrane.org/2013-qu%C3%A9bec-city/assessing-how-much-certainty-place-findings-qualitative-evidence-syntheses-cerqual)
18. Berg, R. C., & Munthe-Kaas, H. (2013). **Systematiske oversikter og kvalitativ forskning**. *Norsk epidemiologi*, *23*(2).
