# Supplementary material for: The use of GRADE-CERQual in qualitative evidence synthesis: an evaluation of fidelity and reporting
Source: Health Res Policy Syst. 2023 Jul 25;21:77. doi: 10.1186/s12961-023-00999-3 (PMC10369711; doi:10.1186/s12961-023-00999-3)
Supplement: Supplementary file 3 — Additional file 3. Coding and charting questions and answers [file 12961_2023_999_MOESM3_ESM.docx]

# **Additional file 3**

## **Coding and charting questions and answers**

### ***Title and abstract coding and charting questions and answers***

| 1. Was a GRADE-GRADE-CERQual coordinating team member a co-author?   - Yes - No - Can’t Tell   2. What language is the publication? (English, Non-English)   - English - In a language other than English - Can’t tell   3. What type of publication is it?   - Review/Synthesis (quant or qual) - Protocol for review (quant or qual) - Primary study - Conference abstract - Methodological or Conceptual paper - Editorial or commentary - Thesis or dissertation - Overview or umbrella review - Other - Can’t tell |
| --- |

### ***Full text coding and charting questions and answers***

| 1. What is the year of publication?  2. Language   - [list of 14 languages] - Other   3. Is GRADE-GRADE-CERQual mentioned in the title, abstract or keywords?   - Yes - No - Can’t tell - Other   4. Was a GRADE-GRADE-CERQual publication referenced in the manuscript and reference list?   - Yes - No - Can’t tell - Other   5. What is the latest guidance authors cite?   - Not applicable, no reference for GRADE-GRADE-CERQual provided - PLOS Med 2015 - Implementation Science Series 2018 - Can't Tell - Other   6. Was GRADE-GRADE-CERQual applied to review findings (interpret "applied" broadly)   - Yes - No - Can’t tell - Other   If you answered "no" to the previous question (i.e. GRADE-GRADE-CERQual was not applied) then respond "not applicable" to all of the remaining questions.  7. If GRADE-GRADE-CERQual was applied, what version of GRADE-GRADE-CERQual was used?   - Not applicable - did not apply GRADE-GRADE-CERQual - 4 component version - 2 component version - Can't tell - Other   8. If GRADE-GRADE-CERQual was applied, what is the Discipline/Field of Study   - Not applicable – did not apply GRADE-GRADE-CERQual - Health - Social Sciences - Education - Environment - Business and Management - Engineering - Agriculture - Arts and Humanities - Can’t tell - Other   9. If GRADE-GRADE-CERQual was applied, what type of review or synthesis was it?   - Not applicable - did not apply GRADE-GRADE-CERQual - Standard systematic review or synthesis - Scoping review - Rapid review - Overview review, umbrella review (review of reviews) - Literature review - Can't tell - Other |
| --- |
