# Supplementary material for: The use of GRADE-CERQual in qualitative evidence synthesis: an evaluation of fidelity and reporting
Source: Health Res Policy Syst. 2023 Jul 25;21:77. doi: 10.1186/s12961-023-00999-3 (PMC10369711; doi:10.1186/s12961-023-00999-3)
Supplement: Supplementary file 4 — Additional file 4. Reporting and fidelity assessment criteria [file 12961_2023_999_MOESM4_ESM.docx]

# **Additional file 4**

## **Reporting and fidelity assessment criteria**

### ***GRADE-CERQual reporting assessment criteria***

| 1. Do authors use the term GRADE-CERQual?   - Yes - Yes, but sometimes use CERQual only - No (they use CERQual only)   2. Is GRADE-CERQual (or CERQual) mentioned in the title, abstract or keywords?   - Yes - No   3. Do the authors use the term CONFIDENCE when defining GRADE-CERQual?   - Yes - Yes, but used interchangeably with other terms (e.g. quality, certainty) - No (they used CERTAINTY, STRENGTH, QUALITY, etc.)   4.1. Is there a table that includes the following 4 elements (review finding, GRADE-CERQual assessment, explanation, references) (in paper or as an additional file)?   - Yes - Yes, but other elements also included (e.g. quote extracts) - Partial (some elements but not others) - No   4.2 Is the table called Summary of Qualitative Findings?   - Yes (exact match) - Yes, but not exact match - No   5.1. Is there a table that contains the following elements (review finding, the assessment and explanation for each of the 4 GRADE-CERQual components, the overall GRADE-CERQual assessment and explanation, references) (in paper or as an additional file)?   - Yes - Yes, but other elements also included (e.g. quote extracts) - Partial (some elements but not others) - No   5.2. Is the table called “Evidence Profile”?   - Yes (exact match) - Yes (but not exact match) - No   6. Is the methodological limitations component named in the manuscript?   - Yes (exact match) - Yes (but not exact match, e.g. name of component slightly changed) - No   7. Is the coherence component named in the manuscript?   - Yes (exact match) - Yes, but not exact match (e.g. name of component slightly changed) - No   8. Is the adequacy (adequacy of data) component named in the manuscript?   - Yes (exact match) - Yes, but not exact match (e.g. name of component slightly changed) - No   9. Is the relevance component named in the manuscript?   - Yes (exact match) - Yes, but not exact match (e.g. name of component slightly changed) - No   10. Are assessments of each component expressed as Concerns (serious, moderate, minor, no or very minor)   - Yes (exact match) - Yes (but not exact match e.g. name of category changed) - Yes (but not all 4 levels mentioned) - No   11. Is an explanation for the assessment of each component provided (not required for no or very minor concerns)?   - Yes - No   12. Is the overall assessment of confidence made using the 4 categories (high, moderate, low, very low)?   - Yes (exact match) - Yes, but not exact match (e.g. name of category changed) - Yes, but not all 4 levels mentioned - No   13. Is an explanation for the overall assessment of confidence provided?   - Yes (minimum) [level of concern per component] - Yes (minimum +) [level of concern per component plus additional explanation] - Yes, but some or all names of components and/or level of concern missing - No   14. Can individual review findings and their GRADE-CERQual assessments be traced back to supporting studies?   - Yes - No |
| --- |

### ***GRADE-CERQual fidelity assessment criteria***

| 1. Authors demonstrate an accurate conceptualisation of GRADE-CERQual (that is an approach for assessing confidence in the individual review findings of a qualitative evidence synthesis).   - No concerns - Concerns   2. Authors have made an overall assessment of confidence based on the assessment of all four components.   - No concerns - Concerns   3. Authors applied GRADE-CERQual to individual review findings   - No concerns - Concerns   4. Authors conceptualise methodological limitations in line with the guidance   - No concerns - Concerns   5. Authors conceptualise coherence in line with the guidance   - No concerns - Concerns   6. Authors conceptualise adequacy in line with the guidance   - No concerns - Concerns   7. Authors conceptualise relevance in line with the guidance   - No concerns - Concerns   8. The GRADE-CERQual assessments are presented in-line with the guidance for SoQF tables and/or Evidence Profiles   - No concerns - Concerns   9. Summary of review findings were produced in-line with the guidance   - No concerns - Concerns |
| --- |
