# Supplementary material for: The use of GRADE-CERQual in qualitative evidence synthesis: an evaluation of fidelity and reporting
Source: Health Res Policy Syst. 2023 Jul 25;21:77. doi: 10.1186/s12961-023-00999-3 (PMC10369711; doi:10.1186/s12961-023-00999-3)
Supplement: Supplementary file 5 — Additional file 5. Results of title and abstract coding and charting [file 12961_2023_999_MOESM5_ESM.docx]

**Additional file 5**

## Results of title and abstract coding and charting (total 1312 studies)

| Title and abstract coding questions | Number of publications  (n=1312) |
| --- | --- |
| Is a coordinating team member a co-author? |  |
| Yes | 135 (10.3%) |
| No | 1112 (84.8%) |
| Can’t tell | 65 (5.0%) |
| Language |  |
| English | 1075 (81.9%) |
| Languages other than English | 237 (18.1%) |
| Type of publication |  |
| Conference abstract | 57 (4.3%) |
| Editorial or Commentary | 33 (2.5%) |
| Methodological or conceptual article | 180 (13.7%) |
| Primary study | 121 (9.2%) |
| Protocol for review (quant or qual) | 129 (9.8%) |
| Thesis or dissertation | 89 (6.8%) |
| Review/synthesis (quant or qual) | 461 (35.1%) |
| Other (including 77 that were the engineering method called “CERQUAL”) | 242 (18.4%) |
