# Supplementary material for: The use of GRADE-CERQual in qualitative evidence synthesis: an evaluation of fidelity and reporting
Source: Health Res Policy Syst. 2023 Jul 25;21:77. doi: 10.1186/s12961-023-00999-3 (PMC10369711; doi:10.1186/s12961-023-00999-3)
Supplement: Supplementary file 6 — Additional file 6. Results of full-text coding and charting that applied GRADE-CERQual [file 12961_2023_999_MOESM6_ESM.docx]

**Additional file 6**

## Results of full-text coding and charting for 233 studies that applied GRADE-CERQual

| Full text coding questions | Number of publications  (n=233) |
| --- | --- |
| Year of Publication |  |
| 2018-August 2020 | 184 (79.0%) |
| 2015-2017 | 49 (21.0%) |
| 2010-2014 | 0 (0.0%) |
| Language |  |
| English | 223 (95.7%) |
| Mandarin | 5 (2.1%) |
| Norwegian | 1 (0.4%) |
| Spanish | 3 (1.3%) |
| Swedish | 1 (0.4%) |
| GRADE-CERQual mentioned in title, abstract or keywords? |  |
| Yes | 102 (43.8%) |
| No | 125 (53.6%) |
| Other (e.g., no abstract) | 6 (%2.6) |
| Was a GRADE-CERQual publication referenced in the manuscript and reference list? |  |
| Yes | 217 (93.1%) |
| No | 4 (1.7%) |
| Other (e.g., referenced a review that applied GRADE-CERQual) | 12 (5.2%) |
| What is the latest guidance authors are citing? |  |
| PLOS Medicine 2015 | 120 (51.5%) |
| Implementation Science Series 2018 | 87 (37.5%) |
| Other (e.g., no reference provided, published review, or other GRADE-CERQual publication cited but not official guidance) | 26 (11.2%) |
| If GRADE-CERQual was applied, what is the Discipline/Field of Study? |  |
| Health | 225 (96.6%) |
| Business and management | 1 (0.4%) |
| Education | 5 (2.1%) |
| Sports science | 1 (0.4%) |
| Social service | 1 (0.4%) |
| If GRADE-CERQual was applied, what type of review or synthesis was it? |  |
| Standard systematic review or synthesis | 223 (95.7%) |
| Scoping review | 3 (1.3%) |
| Overview review/umbrella review | 7 (3.0%) |
