# Supplementary material for: The use of GRADE-CERQual in qualitative evidence synthesis: an evaluation of fidelity and reporting
Source: Health Res Policy Syst. 2023 Jul 25;21:77. doi: 10.1186/s12961-023-00999-3 (PMC10369711; doi:10.1186/s12961-023-00999-3)
Supplement: Supplementary file 7 — Additional file 7. GRADE-CERQual reporting assessment results [file 12961_2023_999_MOESM7_ESM.docx]

**Additional file 7**

## GRADE-CERQual reporting assessment results#

| Reporting Criteria Questions | Number of publications (n=136) |
| --- | --- |
| 1. Do authors use the term GRADE-CERQual? |  |
| Yes | 30 (22.1%) |
| Yes, but sometimes use CERQual only | 61 (44.9%) |
| No (they use CERQual only) | 45 (33.1%) |
| 2. Is GRADE-CERQual (or CERQual) mentioned in the title, abstract or keywords? |  |
| Yes | 82 (60.3%) |
| No | 54 (39.7%) |
| 3. Do the authors use the term CONFIDENCE when defining GRADE-CERQual? |  |
| Yes | 102 (75.0%) |
| Yes, but used interchangeably with other terms (e.g., quality, certainty) | 26 (19.1%) |
| No, they used CERTAINTY, STRENGTH, QUALITY, etc. | 8 (5.9%) |
| 4.1. Is there a table that includes the following 4 elements (review finding, GRADE-CERQual assessment, explanation, references) (in paper or as an additional file)? |  |
| Yes | 46 (33.8%) |
| Yes, but other elements also included (e.g., quote extracts) | 28 (20.6%) |
| Partial (some elements but not others) | 13 (9.6%) |
| No | 49 (36.0%) |
| 4.2 Is the table called Summary of Qualitative Findings? |  |
| Yes (exact match) | 32 (23.5%) |
| Yes, but not exact match | 14 (10.3%) |
| No | 41 (30.1%) |
| Not applicable (no table) | 49 (36.0%) |
| 5.1. Is there a table that contains the following elements (review finding, the assessment and explanation for each of the 4 GRADE-CERQual components, the overall GRADE-CERQual assessment and explanation, references) (in paper or as an additional file)? |  |
| Yes | 71 (52.2%) |
| Yes, but other elements also included (e.g., quote extracts) | 10 (7.4%) |
| Partial (some elements but not others) | 10 (7.4%) |
| No | 45 (33.1%) |
| 5.2. Is the table called “Evidence Profile”? |  |
| Yes (exact match) | 47 (34.6%) |
| Yes (but not exact match) | 10 (7.4%) |
| No | 34 (25.0%) |
| Not applicable | 45 (33.1%) |
| 6. Is the methodological limitations component named in the manuscript? |  |
| Yes (exact match) | 116 (85.3%) |
| Yes (but not exact match, e.g., name of component slightly changed) | 15 (11.0%) |
| No | 5 (3.7%) |
| 7. Is the coherence component named in the manuscript? |  |
| Yes (exact match) | 126 (92.6%) |
| Yes, but not exact match (e.g., name of component slightly changed) | 5 (3.7%) |
| No | 5 (3.7%) |
| 8. Is the adequacy (adequacy of data) component named in the manuscript? |  |
| Yes (exact match) | 125 (91.9%) |
| Yes, but not exact match (e.g., name of component slightly changed) | 4 (2.9%) |
| No | 7 (5.1%) |
| 9. Is the relevance component named in the manuscript? |  |
| Yes (exact match) | 123 (90.4%) |
| Yes, but not exact match (e.g., name of component slightly changed) | 5 (3.7%) |
| No | 8 (5.9%) |
| 10. Are assessments of each component expressed as Concerns (serious, moderate, minor, no or very minor) |  |
| Yes (exact match) | 25 (18.4%) |
| Yes (but not exact match e.g., name of category changed) | 44 (32.4%) |
| Yes (but not all 4 levels mentioned) | 31 (22.8%) |
| No | 36 (26.5%) |
| 11. Is an explanation for the assessment of each component provided (not required for no or very minor concerns)? |  |
| Yes | 92 (67.6%) |
| No | 44 (32.4%) |
| 12. Is the overall assessment of confidence made using the 4 categories (high, moderate, low, very low)? |  |
| Yes (exact match) | 75 (55.1%) |
| Yes, but not exact match (e.g., name of category changed) | 10 (7.4%) |
| Yes, but not all 4 levels mentioned | 45 (33.1%) |
| No | 6 (4.4%) |
| 13. Is an explanation for the overall assessment of confidence provided? |  |
| Yes (minimum) [level of concern per component] | 42 (30.9%) |
| Yes (minimum +) [level of concern per component plus additional explanation] | 5 (3.7%) |
| Yes, but some or all names of components and/or level of concern missing | 67 (49.3%) |
| No | 22 (16.2%) |
| 14. Can individual review findings and their GRADE-CERQual assessments be traced back to supporting studies? |  |
| Yes | 116 (85.3%) |
| No | 20 (14.7%) |

^#^Additional File 8 shows all the sub-codes for responses other than “yes”.
