# Supplementary material for: The use of GRADE-CERQual in qualitative evidence synthesis: an evaluation of fidelity and reporting
Source: Health Res Policy Syst. 2023 Jul 25;21:77. doi: 10.1186/s12961-023-00999-3 (PMC10369711; doi:10.1186/s12961-023-00999-3)
Supplement: Supplementary file 8 — Additional file 8. GRADE-CERQual reporting and fidelity assessment sub-coding of identified concerns by criteria [file 12961_2023_999_MOESM8_ESM.docx]

# **Additional file 8**

## **GRADE-CERQual reporting and fidelity assessment sub-coding of identified concerns by criteria**

### ***Nodes\\Fidelity***

| Name | Files | References |
| --- | --- | --- |
| 1. The authors demonstrate an accurate conceptualization of GRADE-CERQual (that is an approach for assessing confidence in the findings of a qualitative evidence synthesis). | 73 | 155 |
| Appears under quality appraisal section | 10 | 29 |
| Conceptualising it as an assessment of contributing studies | 6 | 13 |
| Define it as a tool for evaluating a review | 1 | 2 |
| Evidence rating tool | 2 | 3 |
| Interchange confidence with certainty | 7 | 14 |
| Interchange confidence with strength | 1 | 1 |
| Not applied to the summarized review findings per se | 3 | 7 |
| Not defined at all | 1 | 1 |
| Referred to as a framework | 5 | 13 |
| Say they use modified version but don't explain | 1 | 4 |
| Scoring system | 2 | 4 |
| Sometimes referred to as a tool to asses quality of findings or evidence | 11 | 29 |
| Their conceptualization is partial (e.g. missing components, no overall assessment, not applied to findings strictly) | 5 | 8 |
| Thought of as a method to reduce bias | 1 | 4 |
| Tool to assess credibility | 1 | 1 |
| Translation issues - unsure | 1 | 1 |
| Use entirely different way of determining overall confidence | 4 | 16 |
| Used to assess explanatory power | 1 | 1 |
| Yes but going beyond and applying in new ways | 1 | 3 |
| 2. The authors have made an overall assessment of confidence based on the assessment of all four components | 31 | 43 |
| No mention of the 4 components at all | 7 | 8 |
| Not clear because merged with GRADE domains | 1 | 2 |
| Some components not assessed | 8 | 11 |
| Adequacy missing | 6 | 8 |
| Coherence missing | 2 | 3 |
| Components are assessed but no overall assessment | 2 | 3 |
| Methodological limitations missing | 1 | 3 |
| Relevance missing | 1 | 1 |
| Unclear as assessments don’t mention all four components | 1 | 2 |
| Yes but assessed each component by study | 1 | 1 |
| Yes but conceptualise components as increasing confidence rather than posing threats to confidence | 1 | 1 |
| Yes but have applied their own scoring rules for determining level of assessment | 7 | 13 |
| 3. The authors applied GRADE-CERQual to individual review findings | 41 | 65 |
| Applied GRADE-CERQual at the study level not finding level | 6 | 10 |
| Applied components to individual findings, but made overall assessment for all findings together | 1 | 3 |
| Applied it to recommendations | 3 | 5 |
| Applied it to short theme or category titles | 14 | 26 |
| Applied to the different elements or propositions of a framework | 3 | 6 |
| Have applied it to codes | 1 | 1 |
| May have applied it to groups of findings | 2 | 2 |
| Unclear, review findings and assessments not obvious | 2 | 3 |
| Yes but do not, or do not appear to have applied to all findings | 4 | 8 |
| Yes but sometimes they say they applied it to individual studies | 2 | 3 |
| 4. Authors conceptualize methodological limitations in line with the guidance | 99 | 243 |
| Applied the levels of concern to individual studies rather than findings | 8 | 16 |
| Authors applied their own criteria for scoring | 2 | 5 |
| Conceptualising the assessment as a count of appraisal categories, not specific limitations in relation to the finding | 28 | 56 |
| Don't define it and no Evidence Profile or SoQf from which to infer | 17 | 20 |
| Go beyond guidance - table just for this component with all concerns by study | 1 | 3 |
| Not conceptualised in terms of Concerns | 14 | 47 |
| Assessments use the categories for overall assessment rather than the levels of concern | 3 | 4 |
| Explanations focus on methodological quality rather than limitations | 3 | 20 |
| Minor or low, moderate, serious or severe methodological limitations, rather than concerns | 3 | 14 |
| Using terms like minor moderate high rather than noting grounds for concern | 5 | 9 |
| Other concerns | 0 | 0 |
| Component only mentioned, no evidence it was assessed | 1 | 2 |
| Defined correctly but unsure because no evidence profile | 1 | 1 |
| Exclude component from assessment on the basis of quality having been addressed earlier in review | 1 | 4 |
| Making an assessment of the whole body of evidence, not evidence supporting individual findings | 2 | 5 |
| No overall judgement for the component provided | 1 | 1 |
| Very different styles of writing the explanations | 1 | 1 |
| Other terminology issues | 0 | 0 |
| Refer to the component as Study Quality | 1 | 1 |
| Use term ‘risk of bias’ to describe this component | 2 | 9 |
| Problems with critical appraisals (e.g. only yes or no, or no explanation) | 20 | 47 |
| Concerns relate to reporting gaps not concrete methodological issues | 3 | 19 |
| Critical appraisals were quantified | 4 | 5 |
| No sign they applied a critical appraisal tool | 1 | 2 |
| Used a reporting tool, not a critical appraisal tool | 3 | 6 |
| Specific methodological limitations mentioned but not how important they are in relation to the finding | 4 | 5 |
| Suspicion due to repetition of the same explanations or assessments across findings | 12 | 18 |
| 5. Authors conceptualize coherence in line with the guidance | 126 | 234 |
| Don't define it and no Evidence Profile or SoQf from which to infer | 23 | 24 |
| Go beyond guidance - table just for this component with all concerns by study | 1 | 2 |
| No demonstration of thinking of it in terms of the fit between review finding and data from primary studies, only focus on primary studies | 15 | 24 |
| Not conceptualised in terms of Concerns | 21 | 30 |
| Applied the levels of concern to individual studies rather than findings | 6 | 7 |
| Assessment uses term high coherence rather than levels of concern | 14 | 21 |
| Looking for proof of coherence, rather than concerns about coherence | 1 | 2 |
| Other concerns | 10 | 23 |
| Component never mentioned | 2 | 2 |
| Component only mentioned, no evidence it was assessed | 1 | 2 |
| Defined correctly but don't explain the concern about the fit between review finding and study findings | 2 | 2 |
| Explanations for assessments not about coherence | 2 | 3 |
| Lack of consistency in how explanations written, as though different authors | 2 | 12 |
| May have written findings in a way to ensure high coherence | 1 | 2 |
| Problems of definition | 46 | 82 |
| Defined as an assessment of whether clear patterns could be assessed across studies | 2 | 2 |
| Defined as consistency or agreement within and across studies | 34 | 63 |
| Definition and explanations not related to coherence | 2 | 4 |
| Definition given partial or incomplete | 1 | 1 |
| Old definition from 2015 | 9 | 12 |
| Quantification of the assessment (counting) | 8 | 21 |
| Reference to issues more related to relevance | 6 | 9 |
| Suspicions because the same assessment or explanation is repeated across findings | 3 | 3 |
| 6. Authors conceptualize adequacy of data in line with the guidance | 118 | 217 |
| Assessed level of concern at the individual study level not review finding level | 4 | 5 |
| Bring other concepts into their conceptualization of adequacy | 5 | 19 |
| Claim adequacy is similar to the concept of data saturation | 2 | 8 |
| Gravitational pull | 1 | 7 |
| They consider saturation and member checking in addition to richness and quantity | 2 | 4 |
| Don't define it and no Evidence Profile or SoQf from which to infer | 18 | 19 |
| Go beyond the guidance - table for just this component | 1 | 1 |
| Not assessed in terms of Concerns | 12 | 36 |
| Apply the terminology for the overall assessment to the component assessment | 2 | 8 |
| Assessment uses term high, moderate, minor rather than levels of concern | 7 | 11 |
| Conceptualising it in terms of assessing whether adequacy has been achieved rather than noting concerns | 4 | 17 |
| Not assessing both quantity and richness, emphasizing one or the other | 18 | 27 |
| Focus on quantity (number of studies or sample size) not richness | 11 | 20 |
| Focus on richness, nothing about quantity | 7 | 7 |
| Other concerns | 0 | 0 |
| Component left-out of the evidence profile | 2 | 3 |
| Definition correct but without tables or enough detail not possible to assess conceptualisation fully | 2 | 2 |
| Definition provided could have been worded better | 1 | 1 |
| Misnamed sufficiency | 2 | 3 |
| No mention of component at all | 3 | 4 |
| Probably human error (mixing-up definitions, using quality instead of quantity) | 2 | 2 |
| Suspicions because the same level of concern or explanations are repeated | 1 | 1 |
| Variability in how explanations written, as though different authors | 1 | 1 |
| Overlap with other components | 0 | 0 |
| Confounding with coherence | 2 | 2 |
| Confounding with methodological limitations (refer to sampling, saturation) | 3 | 3 |
| Confounding with Relevance (mostly by emphasizing range of countries) | 20 | 27 |
| Settings, type of study, intervention | 1 | 1 |
| Quantify the assessment of the component | 11 | 32 |
| Conceptualised as quantity of findings in the primary studies | 3 | 3 |
| Conceptualising the assessment as a count of studies, not in relation to the finding | 6 | 9 |
| 7. Authors conceptualize relevance in line with the guidance | 121 | 224 |
| Deliberately exclude this component from the GRADE-CERQual approach | 2 | 2 |
| Direct, indirect, partial not used or incorrectly used | 18 | 27 |
| Do not use recommended terminology of indirect, partial, unclear | 18 | 25 |
| Use dichotomy - relevant, not relevant | 1 | 2 |
| Go beyond the guidance - a table just for this component's assessments | 1 | 1 |
| Issues of definition | 32 | 55 |
| Definition is missing clear reference to the review question | 3 | 4 |
| Deliberate modification of definition | 0 | 0 |
| Relevance of the concepts rather than relevance of the studies | 1 | 7 |
| Theme's presence across timeframes | 1 | 1 |
| Don't define it and no EP or SoQf from which to infer | 20 | 21 |
| Refer to relevance of findings rather than relevance of study | 4 | 14 |
| Refer to review objectives rather than review question | 3 | 14 |
| They use but don't define partial, indirect, unclear | 2 | 2 |
| Language of concerns not used, or not used correctly | 27 | 54 |
| Applied language of concerns to individual studies | 2 | 3 |
| Apply language of overall assessment to individual component assessment | 1 | 4 |
| Assessment uses terms high, moderate, minor rather than levels of concern | 15 | 20 |
| Assessments not expressed as concerns | 9 | 27 |
| Not all elements of context were considered in the assessment | 34 | 59 |
| Assessment limited to aim, topic, phenomenon of interest only | 12 | 23 |
| Assessment limited to population only | 3 | 3 |
| Assessment limited to setting in terms of geography (country, high or low income country, urban or rural) | 16 | 30 |
| Refer to country only | 13 | 26 |
| Assessment limited to settings and phenomenon of interest or intervention | 2 | 2 |
| Assessment limited to topic and population | 1 | 1 |
| Other concerns | 12 | 20 |
| Assessing relevance for the whole of included studies in review, not specific to finding | 1 | 2 |
| Claim they excluded non relevant studies | 2 | 4 |
| Component never mentioned | 1 | 1 |
| Correct definition but no tables or inadequate info from which to evaluate their conceptualisation in practice | 2 | 2 |
| Relevance very infrequently or never referred to in the explanation for their overall assessments | 3 | 4 |
| Suspicions because the same assessment or explanation repeated across findings | 3 | 5 |
| Variability in how explanations written, possibly different authors | 2 | 2 |
| Overlap with other components | 2 | 3 |
| Confounding with coherence | 1 | 1 |
| Confounding with methodological limitations | 1 | 2 |
| Quantify the assessment by counting how many primary studies are indirect or partial, not using concerns | 9 | 22 |
| 8. The GRADE-CERQual assessments are presented in-line with the guidance for SoQf tables and or Evidence Profiles | 94 | 114 |
| Additional information included | 25 | 30 |
| Added column or info - Importance | 2 | 2 |
| Added column or info - Phenomenon of Interest and Outcome | 1 | 1 |
| Added column or info - add the total number of studies supporting the finding | 1 | 1 |
| Added column or info - Emergent findings incorporated into synthesized finding | 1 | 1 |
| Added column or info - illustrative quotes | 12 | 13 |
| Added column or info - Method or Study Design | 3 | 3 |
| Added column or info - number of studies per country, countries, setting | 1 | 1 |
| Added column or info - Recommendations | 1 | 1 |
| Added column or info - Theme(s) | 4 | 4 |
| Added column or info - Theory | 2 | 2 |
| Added column or info- behaviour change techniques that relate to recommendation | 1 | 1 |
| Issues with presentation of the explanations for component or overall assessment | 25 | 31 |
| Errors or modifications in terminology (concern levels, confidence levels, which ones to use where) | 14 | 16 |
| Explanation for the overall assessment does not follow guidance | 6 | 6 |
| Explanations appear in footnotes | 5 | 6 |
| Explanations for the component assessments are added to the overall assessment explanation column | 3 | 3 |
| Key elements missing or left out | 38 | 55 |
| Explanation for the overall assessment missing from SoQf and or Evidence Profile | 23 | 26 |
| No summaries of review findings in tables | 0 | 0 |
| No summaries of review findings - codes and descriptions instead | 1 | 1 |
| No summaries of review findings - just study design | 1 | 1 |
| No summaries of review findings - propositions that make-up a framework | 1 | 1 |
| No summaries of review findings - recommendations | 2 | 3 |
| No summaries of review findings - themes and sub-themes | 17 | 18 |
| Number of supporting studies reported but no references | 6 | 7 |
| One component missing | 3 | 4 |
| Overall assessment is missing | 2 | 2 |
| References for supporting studies are missing | 7 | 8 |
| Some or all explanations for component assessments are missing | 7 | 8 |
| No SoQf or Evidence Profile included | 9 | 9 |
| GRADE-CERQual not applied to all review findings | 1 | 3 |
| Other formatting divergences | 13 | 23 |
| Looks like they are following an EPOC template in terms of table design | 1 | 1 |
| Multiple columns combined into one | 3 | 3 |
| Multiple SoQfs - one per theme | 1 | 6 |
| Not presented as a table but rather as a box with headings and bullet points | 1 | 1 |
| Relevance and or Adequacy given two columns each | 2 | 4 |
| Tables includes findings from other types of syntheses (e.g. quant) | 3 | 3 |
| They adopted the little symbols GRADE uses for the overall assessment | 4 | 5 |
| 9. Summarized review findings were produced in line with the guidance | 81 | 100 |
| Length of findings | 12 | 12 |
| Relatively long findings | 2 | 2 |
| Relatively short findings | 10 | 10 |
| No summary of review findings because no tables | 5 | 5 |
| Not review findings as we have defined them | 0 | 0 |
| Just theme or category names, not summarized review findings | 34 | 38 |
| Not review findings but propositions that make up their framework | 1 | 1 |
| Not review findings, but rather code definitions | 1 | 1 |
| Not review findings, but rather recommendations | 2 | 3 |
| Theme names followed by bullet points | 1 | 1 |
| Summarized review findings only appear in Evidence Profile, SoQf has short themes or vis versa | 5 | 5 |
| They provide summary statements but GRADE-CERQual not applied to these, just to overall theme | 1 | 1 |

### ***Nodes\\Reporting***

| Name | Files | References |
| --- | --- | --- |
| a. Do authors use the term GRADE-CERQual | 0 | 0 |
| No (they use GRADE-CERQual only) | 53 | 81 |
| Yes (but sometimes use GRADE-CERQual only) | 71 | 135 |
| b. Is GRADE-CERQual or GRADE-CERQual mentioned in the title, abstract or keywords | 0 | 0 |
| c. Do authors use the term CONFIDENCE when defining GRADE-CERQual | 0 | 0 |
| No (e.g. they used e.g. CERTAINTY, STRENGTH, QUALITY ) | 8 | 17 |
| Certainty | 4 | 8 |
| Explanatory Power | 1 | 1 |
| Quality | 4 | 9 |
| Yes but used interchangeably with other terms (e.g. quality, certainty) | 36 | 74 |
| Certainty | 13 | 23 |
| Credibility | 1 | 1 |
| Quality | 16 | 24 |
| Strength of evidence | 7 | 7 |
| d.1. Is there a table that contains the following four following elements (review finding, GRADE-CERQual assessment, explanation, references) (in paper or as an additional file) | 0 | 0 |
| Partial (some elements but not others) | 16 | 21 |
| Missing explanation of overall assessment | 10 | 10 |
| Missing overall assessment of confidence | 1 | 1 |
| Missing references | 9 | 9 |
| Yes but other elements also included (e.g. quote extracts) | 25 | 27 |
| Comments | 1 | 1 |
| Emergent findings synthesised by review finding | 1 | 1 |
| Method or study design | 3 | 3 |
| Number of participants | 1 | 1 |
| Number of studies | 3 | 3 |
| Objective | 5 | 5 |
| Perspective (e.g. mothers, providers) | 5 | 5 |
| Phenomenon of interest or intervention | 1 | 1 |
| Rating symbols | 1 | 1 |
| Relevant theories | 1 | 1 |
| Supporting Quotes | 8 | 8 |
| Third and or second order themes | 1 | 1 |
| d.2. Is the table called “Summary of Qualitative Findings” | 0 | 0 |
| No | 45 | 46 |
| Called Evidence Profile | 6 | 6 |
| Varied titles but that don't name GRADE-CERQual but refer to summaries of findings, themes or recommendations | 7 | 7 |
| Varied titles but that include GRADE-CERQual, GRADE- CERQual or confidence assessments | 25 | 25 |
| Varied titles that refer to confidence, strength, certainty, evidence without naming GRADE-CERQual | 6 | 6 |
| Yes, but not exact match | 23 | 25 |
| Addition of the word Review in front of findings | 2 | 2 |
| Includes addition of GRADE-CERQual or confidence assessment in title | 12 | 12 |
| Missing the word qualitative - summary of findings | 15 | 17 |
| Summary of Themes rather Summary of Findings | 1 | 1 |
| e.1. Is there a table that contains the following elements (review finding, the assessment and explanation for each of the 4 GRADE-CERQual components, the overall GRADE-CERQual assessment and explanation, references) | 0 | 0 |
| Partial (some elements but not others) | 12 | 13 |
| No explanation for component assessments | 1 | 1 |
| No explanation for overall assessment | 11 | 11 |
| No overall assessment | 2 | 2 |
| One or more components not assessed | 5 | 5 |
| Yes but other elements also included (e.g. quote extracts) | 13 | 18 |
| Number of studies or participants contributing to finding | 5 | 5 |
| Overall importance of the finding | 2 | 2 |
| Quality assessment | 1 | 1 |
| Studies contradicting the review finding | 1 | 1 |
| Study context (country, setting, population) | 2 | 2 |
| Study design | 5 | 5 |
| Supporting Quotes | 2 | 2 |
| Theme name to which the review finding pertains | 4 | 4 |
| Two columns for some components | 2 | 2 |
| e.2. Is the table called “Evidence Profile” | 0 | 0 |
| No | 40 | 41 |
| Called Summary of (qualitative) Findings | 6 | 6 |
| No title for table provided | 1 | 1 |
| Other | 2 | 2 |
| Varied titles but that include CERQual, GRADE-CERQual or confidence assessments | 28 | 28 |
| Varied titles that refer to confidence, strength, certainty, evidence without naming GRADE-CERQual | 3 | 3 |
| Yes, but not exact match | 10 | 10 |
| f. Is the methodological limitations component named in the manuscript or tables | 0 | 0 |
| No | 1 | 1 |
| Not named explicitly | 1 | 1 |
| Yes (but not exact match, e.g. name of component slightly changed) | 20 | 25 |
| Can't tell | 2 | 2 |
| methodologic limitations | 1 | 1 |
| Methodological assessment | 1 | 1 |
| methodological concern | 1 | 1 |
| Methodological issues | 1 | 1 |
| methodological quality | 10 | 10 |
| Methodological rigor | 1 | 1 |
| Methods | 1 | 1 |
| study quality | 1 | 1 |
| g. Is the coherence component named in the manuscript or tables | 0 | 0 |
| No | 1 | 1 |
| Not explicitly named | 1 | 1 |
| Yes (but not exact match, e.g. name of component slightly changed) | 4 | 5 |
| Can't tell | 2 | 2 |
| Cohesion | 2 | 2 |
| h. Is the relevance component named in the manuscript or tables | 0 | 0 |
| No | 2 | 2 |
| Not explicitly named | 2 | 2 |
| Yes (but not exact match, e.g. name of component slightly changed) | 3 | 4 |
| Applicability | 2 | 2 |
| Can't tell | 1 | 1 |
| Relevancy | 1 | 1 |
| i. Is the adequacy (or adequacy of data) component named in the manuscript | 0 | 0 |
| No | 1 | 1 |
| Not explicitly named | 1 | 1 |
| Yes (but not exact match, e.g. name of component slightly changed) | 5 | 7 |
| Adequacy-Richness of Evidence | 1 | 1 |
| Can't tell | 1 | 1 |
| data adequacy | 1 | 1 |
| Interchangeably with adequacy of evidence | 2 | 2 |
| j. Are assessments of each component expressed as Concerns (serious, moderate, minor, no or very minor) | 0 | 0 |
| No | 35 | 51 |
| Explanation without a level of assessment | 10 | 10 |
| High instead of serious | 2 | 2 |
| Medium instead of moderate | 1 | 1 |
| Minimal instead of minor | 1 | 1 |
| None, low, medium | 1 | 1 |
| Numbers | 5 | 5 |
| Serious (not serious, serious, very serious) | 2 | 2 |
| Substantial or significant instead of serious | 3 | 3 |
| Use of high, moderate low, minor but not the word concerns | 10 | 10 |
| Yes (but not all 4 levels mentioned) | 26 | 32 |
| Yes (but not exact match) | 54 | 63 |
| High instead of no concerns | 1 | 2 |
| Low instead of minor | 2 | 5 |
| Major concerns | 8 | 9 |
| Mild instead of minor | 1 | 2 |
| Minimal instead of very minor | 1 | 2 |
| Minor to Moderate | 2 | 2 |
| Moderate to serious concerns | 1 | 1 |
| No instead of no or very minor or vis versa | 28 | 37 |
| No issues instead of no concerns | 1 | 2 |
| No to minor instead of no to very minor | 1 | 1 |
| None instead of no | 1 | 1 |
| Significant instead of serious | 1 | 1 |
| Substantial concerns | 21 | 23 |
| k. Is an explanation for the assessment of each component provided (not required for no or very minor concerns) | 0 | 0 |
| No | 11 | 21 |
| l. Is the overall assessment of confidence made using the 4 categories (high, moderate, low, very low) | 0 | 0 |
| No | 6 | 7 |
| Did not do an overall assessment | 3 | 3 |
| only 3 categories (left-out very low) | 4 | 4 |
| They use level of concern | 1 | 1 |
| Yes (but not all 4 levels mentioned) | 49 | 83 |
| Yes (but not exact match, e.g. name of category changed) | 13 | 14 |
| High to moderate (or vis-versa) | 2 | 2 |
| Low to moderate (or vis-versa) | 3 | 3 |
| Medium instead of moderate | 5 | 7 |
| Missing word confidence, just high, low etc | 1 | 1 |
| Quite low instead of very low | 1 | 1 |
| m. Is an explanation for the overall assessment of confidence provided | 0 | 0 |
| No | 12 | 14 |
| Yes (but some or all names of components and-or level of concern missing) | 70 | 97 |
| Yes (minimum +) | 8 | 8 |
| Yes (minimum) | 29 | 31 |
| n. Can individual review findings and their GRADE-CERQual assessments be traced back to supporting studies | 0 | 0 |
| No | 18 | 19 |
