# Supplementary material for: The use of GRADE-CERQual in qualitative evidence synthesis: an evaluation of fidelity and reporting
Source: Health Res Policy Syst. 2023 Jul 25;21:77. doi: 10.1186/s12961-023-00999-3 (PMC10369711; doi:10.1186/s12961-023-00999-3)
Supplement: Supplementary file 9 — Additional file 9. Issues specific to each component [file 12961_2023_999_MOESM9_ESM.docx]

**Additional file 9**

## Issues specific to each component (in full)

GRADE-CERQual approach has four components (methodological limitations, coherence, relevance, and adequacy of data) and the evaluation identified the following component-specific issues.

***Lack of required detail for assessing Methodological Limitations.*** A particular concern in relation to the component methodological limitations was, as already mentioned, that assessments were often limited to counting how many studies had limitations but not mentioning what those limitations were, nor demonstrating that specific limitations were considered in relation to the individual review findings. A limitation in a study might not affect our confidence in all findings. We may be less concerned about some limitations if we think that limitation was unlikely to affect the findings that contributed to the review finding. To properly reflect on this at the stage of applying GRADE-CERQual, the reviewers need critical appraisal tables with more than just a “yes” or a “no” answer for each appraisal question. When the response is no, the reason should be described. It is these specific limitations that the reviewers must consider in relation to the specific review finding when assessing methodological limitations.

***Coherence conceptualised differently than in guidance.*** Of all 9 fidelity questions, concerns about the way authors conceptualised the coherence component were the most common. 31 studies defined coherence very similarly as “consistency within and across studies”. We consider this a serious fidelity concern, because it misses a key aspect of the component’s definition in the current guidance, that is, that it is the assessment of fit between the findings in the primary studies *and the review finding*. “Consistent within and across studies” makes no reference to the review finding at all. In the guidance coherence is defined as “an assessment of how clear and cogent the fit is between the data from the primary studies and a review finding that synthesises that data” (16). To assess coherence, we are not just comparing data between contributing studies, but data from the contributing studies and the review finding as we have written it. It is not entirely clear where “consistency within and across studies” emerged, but it is uncanny how consistently (no pun intended) it appeared across studies. The coherence component was the only component to significantly change in definition between the 2015 guidance and the 2018 guidance. However, the 2015 definition (12) also does not include the word consistency. Consistency instead is mentioned in relation to GRADE, specifically the inconsistency domain of the GRADE approach. It is possible that the notion of consistency within and across studies is a hangover from the GRADE approach which some reviewers may have been used to applying in the context of quantitative systematic reviews.

***Assessing some but not all aspects of Relevance and Adequacy.*** Both these components include more than one aspect for reviewers to consider. In the case of adequacy this is both “richness” and “quantity” of data. We noted in 16 reviews that reviewers were either emphasising richness or quantity in their assessments of this component, but not both. This is an important fidelity concern because both aspects are fundamental to the concept of adequacy. Relevance is defined as “the extent to which the body of data from the primary studies supporting a review finding is applicable to the context specified in the review question” (18). Importantly, there are many aspects of “context”, including the perspective or population, phenomenon of interest, and setting. In 31 reviews, we found evidence that authors were thinking about one, but not all these aspects of context. The most common interpretation of “context” was country, followed by topic/phenomenon of interest.

## **The boundary between components**

Mutually exclusive definitions of each component have been a challenge, as there is natural overlap between the conceptual basis of each component. This evaluation gave us the opportunity to identify if and how authors confound components, by, for example, considering aspects in their assessment of one component, that, based on the guidance, relate to another. For example, in assessments of coherence, issues relating to relevance such as perspective, phenomenon of interest, population, or settings were sometimes referenced in the explanation of the assessment. For adequacy of data, we noted a couple examples of mentioning issues relating to coherence (e.g. contradictory data) and methodological limitations (issues around reflexivity). Much more common was mention of issues overlapping with relevance (population, setting). As for relevance, on only two occasions did we note that explanations overlapped with either methodological limitations (issues around information given to participants) or coherence (coherent picture).
